# Genetically proxied therapeutic inhibition of antihypertensive drug targets and risk of common cancers: A mendelian randomization analysis

**DOI:** 10.1371/journal.pmed.1003897

**Published:** 2022-02-03

**Authors:** James Yarmolinsky, Virginia Díez-Obrero, Tom G. Richardson, Marie Pigeyre, Jennifer Sjaarda, Guillaume Paré, Venexia M. Walker, Emma E. Vincent, Vanessa Y. Tan, Mireia Obón-Santacana, Demetrius Albanes, Jochen Hampe, Andrea Gsur, Heather Hampel, Rish K. Pai, Mark Jenkins, Steven Gallinger, Graham Casey, Wei Zheng, Christopher I. Amos, the International Lung Cancer Consortium, the PRACTICAL consortium, the MEGASTROKE consortium, George Davey Smith, Richard M. Martin, Victor Moreno

**Affiliations:** 1 MRC Integrative Epidemiology Unit, University of Bristol, Bristol, United Kingdom; 2 Population Health Sciences, Bristol Medical School, University of Bristol, Bristol, United Kingdom; 3 Biomarkers and Susceptibility Unit, Oncology Data Analytics Program, Catalan Institute of Oncology (ICO), L’Hospitalet de Llobregat, Barcelona, Spain; 4 Colorectal Cancer Group, ONCOBELL Program, Bellvitge Biomedical Research Institute (IDIBELL), L’Hospitalet de Llobregat, Barcelona, Spain; 5 Consortium for Biomedical Research in Epidemiology and Public Health (CIBERESP), Madrid, Spain; 6 Population Health Research Institute, David Braley Cardiac, Vascular and Stroke Research Institute, Hamilton, Canada; 7 Thrombosis and Atherosclerosis Research Institute, David Braley Cardiac, Vascular and Stroke Research Institute, Hamilton, Canada; 8 Department of Medicine, Michael G. DeGroote School of Medicine, McMaster University, Hamilton, Canada; 9 Department of Pathology and Molecular Medicine, McMaster University, Michael G. DeGroote School of Medicine, Hamilton, Canada; 10 Department of Clinical Epidemiology and Biostatistics, McMaster University, Ontario, Canada; 11 Department of Surgery, University of Pennsylvania Perelman School of Medicine, Philadelphia, Pennsylvania, United States of America; 12 School of Cellular and Molecular Medicine, University of Bristol, Bristol, United Kingdom; 13 Division of Cancer Epidemiology and Genetics, National Cancer Institute, National Institutes of Health, Bethesda, Maryland, United States of America; 14 Department of Medicine I, University Hospital Dresden, Technische Universität Dresden (TU Dresden), Dresden, Germany; 15 Institute of Cancer Research, Department of Medicine I, Medical University Vienna, Vienna, Austria; 16 Division of Human Genetics, Department of Internal Medicine, The Ohio State University Comprehensive Cancer Center, Columbus, Ohio, United States of America; 17 Department of Laboratory Medicine and Pathology, Mayo Clinic Arizona, Scottsdale, Arizona, United States of America; 18 Centre for Epidemiology and Biostatistics, The University of Melbourne, Parkville, Australia; 19 Division of General Surgery, University Health Network, University of Toronto, Toronto, Canada; 20 Center for Public Health Genomics and Department of Public Health Sciences, University of Virginia, Charlottesville, Virginia, United States of America; 21 Division of Epidemiology, Vanderbilt University Medical Center, Vanderbilt University, Nashville, Tennessee, United States of America; 22 Department of Medicine, Baylor College of Medicine, Institute for Clinical and Translational Research, Houston, Texas, United States of America; 23 University Hospitals Bristol, NHS Foundation Trust, National Institute for Health Research Bristol Biomedical Research Centre, University of Bristol, Bristol, United Kingdom; 24 Department of Clinical Sciences, Faculty of Medicine, University of Barcelona, Barcelona, Spain; Imperial College London, UNITED KINGDOM

## Abstract

**Background:**

Epidemiological studies have reported conflicting findings on the potential adverse effects of long-term antihypertensive medication use on cancer risk. Naturally occurring variation in genes encoding antihypertensive drug targets can be used as proxies for these targets to examine the effect of their long-term therapeutic inhibition on disease outcomes.

**Methods and findings:**

We performed a mendelian randomization analysis to examine the association between genetically proxied inhibition of 3 antihypertensive drug targets and risk of 4 common cancers (breast, colorectal, lung, and prostate). Single-nucleotide polymorphisms (SNPs) in *ACE*, *ADRB1*, and *SLC12A3* associated (*P* < 5.0 × 10^−8^) with systolic blood pressure (SBP) in genome-wide association studies (GWAS) were used to proxy inhibition of angiotensin-converting enzyme (ACE), β-1 adrenergic receptor (ADRB1), and sodium-chloride symporter (NCC), respectively. Summary genetic association estimates for these SNPs were obtained from GWAS consortia for the following cancers: breast (122,977 cases, 105,974 controls), colorectal (58,221 cases, 67,694 controls), lung (29,266 cases, 56,450 controls), and prostate (79,148 cases, 61,106 controls). Replication analyses were performed in the FinnGen consortium (1,573 colorectal cancer cases, 120,006 controls). Cancer GWAS and FinnGen consortia data were restricted to individuals of European ancestry. Inverse-variance weighted random-effects models were used to examine associations between genetically proxied inhibition of these drug targets and risk of cancer. Multivariable mendelian randomization and colocalization analyses were employed to examine robustness of findings to violations of mendelian randomization assumptions. Genetically proxied ACE inhibition equivalent to a 1-mm Hg reduction in SBP was associated with increased odds of colorectal cancer (odds ratio (OR) 1.13, 95% CI 1.06 to 1.22; *P* = 3.6 × 10^−4^). This finding was replicated in the FinnGen consortium (OR 1.40, 95% CI 1.02 to 1.92; *P* = 0.035). There was little evidence of association of genetically proxied ACE inhibition with risk of breast cancer (OR 0.98, 95% CI 0.94 to 1.02, *P* = 0.35), lung cancer (OR 1.01, 95% CI 0.92 to 1.10; *P* = 0.93), or prostate cancer (OR 1.06, 95% CI 0.99 to 1.13; *P* = 0.08). Genetically proxied inhibition of ADRB1 and NCC were not associated with risk of these cancers. The primary limitations of this analysis include the modest statistical power for analyses of drug targets in relation to some less common histological subtypes of cancers examined and the restriction of the majority of analyses to participants of European ancestry.

**Conclusions:**

In this study, we observed that genetically proxied long-term ACE inhibition was associated with an increased risk of colorectal cancer, warranting comprehensive evaluation of the safety profiles of ACE inhibitors in clinical trials with adequate follow-up. There was little evidence to support associations across other drug target–cancer risk analyses, consistent with findings from short-term randomized controlled trials for these medications.

## Abstract 2

In a Mendelian randomization analysis, James Yarmolinsky and colleagues investigate associations between genetically-proxied inhibition of antihypertensive drug targets and breast, colorectal, lung, and prostate cancer risk.

## Author summary

**Why was this study done?:**

- Angiotensin-converting enzyme (ACE) inhibitors, beta blockers, and thiazide diuretics are commonly prescribed antihypertensive medications.
- Some epidemiological studies have suggested that long-term use of these medications can increase cancer risk, though findings have been conflicting.
- Germline genetic variation in genes encoding drug targets can be used to proxy the effect of long-term modulation of these targets on disease endpoints (“drug-target mendelian randomization”).

**What did the researchers do and find?:**

- We used drug-target mendelian randomization to examine the association of genetically proxied inhibition of the drug targets of ACE inhibitors, beta blockers, and thiazide diuretics with risk of 4 of the most common adult cancers (breast, colorectal, lung, and prostate) in up to 289,612 cancer cases and 291,224 controls.
- We found evidence that genetically proxied inhibition of the drug target for ACE inhibitors was associated with an increased risk of colorectal cancer.
- There was little evidence that genetically proxied inhibition of the drug target for ACE inhibitors was associated with risk of the other cancers examined or evidence for an association of genetically proxied inhibition of drug targets for beta blockers and thiazide diuretics with risk of all 4 cancers examined.

**What do these findings mean?:**

- These findings provide support for a link between long-term inhibition of the drug target for ACE inhibitors and colorectal cancer risk, highlighting the need to evaluate the safety profiles of these medications in clinical trials with adequate follow-up length.
- Prior to confirmation of an effect of ACE inhibitor use on colorectal cancer risk in clinical trials, these findings should not alter clinical practice for ACE inhibitor prescribing.

## Introduction

Angiotensin-converting enzyme (ACE) inhibitors are commonly prescribed antihypertensive medications [1]. These medications lower blood pressure by inhibiting the conversion of angiotensin I to angiotensin II, a vasoconstrictor and the primary effector molecule of the renin–angiotensin system (RAS). Though clinical trials have supported the relative safety of these medications in the short term (median follow-up of 3.5 years), concerns have been raised that long-term use of these medications could increase risk of cancer [2,3]. These safety concerns relate to the multifaceted role of ACE, which cleaves various other substrates beyond angiotensin I, including several peptides that have proliferative effects. For example, ACE inhibition leads to the accumulation of bradykinin, an inflammatory mediator involved in tumor growth and metastasis [4]. In addition, substance P is elevated in ACE inhibitor users, which can promote tumor proliferation, migration, and angiogenesis [5,6].

Some observational epidemiological studies have suggested potential adverse effects of long-term use of these drugs on risk of common cancers (i.e., breast, colorectal, lung, and prostate) [7–10], though findings have been largely inconsistent (i.e., null and protective associations have also been reported for the relationship between ACE inhibitor use and cancer risk) [11–15]. Interpretation of the epidemiological literature is challenging for several reasons. First, pharmaco-epidemiological studies are susceptible to residual confounding due to unmeasured or imprecisely measured confounders, including those related to indication [16]. Second, several studies examining ACE inhibitor use and cancer risk have included prevalent drug users, which can introduce bias because prevalent users are “survivors” of the early period of pharmacotherapy and because covariates at study entry can be influenced by prior medication use [12,17–20]. Third, some prior studies may have suffered from time-related biases, including immortal time bias, which can arise because of misalignment of the start of follow-up, eligibility, and treatment assignment of participants [17,18,20,21]. These biases can produce illusory results in favor of the treatment group, while other biases often pervasive in the pharmaco-epidemiological literature (e.g., detection bias due to more intensive clinical monitoring and testing of individuals receiving treatment) can alternatively generate upward-biased effect estimates among those receiving treatment.

Along with ACE inhibitors, β blockers and thiazide diuretics are commonly prescribed antihypertensive medications that lower blood pressure through pathways independent to that of ACE (i.e., β blockers bind to β-adrenergic receptors, inhibiting the binding of norepinephrine and epinephrine to these receptors; thiazide diuretics promote sodium and water excretion by inhibiting sodium reabsorption in renal tubules) [4]. Some in vitro and epidemiological studies have suggested potential chemopreventive effects of these medications on cancer risk, though findings have been inconclusive [22–30].

Naturally occurring variation in genes encoding antihypertensive drug targets can be used as proxies for these targets to examine the effect of their therapeutic inhibition on disease outcomes (“mendelian randomization”) [31,32]. Such an approach should be less prone to conventional issues of confounding as germline genetic variants are randomly assorted at meiosis. In addition, mendelian randomization analysis permits the effect of long-term modulation of drug targets on cancer risk to be examined. Drug-target mendelian randomization can therefore be used to mimic the effect of pharmacologically modulating a drug target in clinical trials and has been used previously to anticipate clinical benefits and adverse effects of therapeutic interventions [33–36].

We used a mendelian randomization approach to examine the effect of long-term inhibition of the drug targets for ACE inhibitors (ACE; angiotensin-converting enzyme), β blockers (ADRB1; beta-1 adrenergic receptor), and thiazide diuretic agents (NCC; sodium-chloride symporter) on risk of overall and subtype-specific breast, colorectal, lung, and prostate cancer.

## Methods

### Study populations

For primary analyses, summary genetic association data were obtained from 4 cancer genome-wide association study (GWAS) consortia. Summary genetic association estimates for overall and estrogen receptor (ER)–stratified breast cancer risk in up to 122,977 cases and 105,974 controls were obtained from the Breast Cancer Association Consortium (BCAC) [37]. Summary genetic association estimates for overall and site-specific colorectal cancer risk in up to 58,221 cases and 67,694 controls were obtained from an analysis of the Genetics and Epidemiology of Colorectal Cancer Consortium (GECCO), ColoRectal Transdisciplinary Study (CORECT), and Colon Cancer Family Registry (CCFR) [38]. Summary genetic association estimates for overall and histological subtype-stratified lung cancer risk in up to 29,266 cases and 56,450 controls were obtained from an analysis of the Integrative Analysis of Lung Cancer Risk and Etiology (INTEGRAL) team of the International Lung Cancer Consortium (ILCCO) [39]. Summary genetic association estimates for overall and advanced prostate cancer risk in up to 79,148 cases and 61,106 controls were obtained from the Prostate Cancer Association Group to Investigate Cancer Associated Alterations in the Genome (PRACTICAL) consortium [40]. These analyses were restricted to participants of European ancestry.

For replication analyses, summary genetic association data were obtained on 1,573 colorectal cancer cases and 120,006 controls of European ancestry from the Finngen consortium. We also examined whether findings could be extended to individuals of East Asian ancestry by obtaining summary genetic association data on 23,572 colorectal cancer cases and 48,700 controls of East Asian ancestry from a GWAS meta-analysis of the Asia Colorectal Cancer Consortium and the Korean National Cancer Center CRC Study 2 [41].

Further information on statistical analysis, imputation, and quality control measures for these studies is available in the original publications. All studies contributing data to these analyses had the relevant institutional review board approval from each country, in accordance with the Declaration of Helsinki, and all participants provided informed consent.

### Instrument construction

To generate instruments to proxy ACE, ADRB1, and NCC inhibition, we pooled summary genetic association data from 2 previously published GWAS of systolic blood pressure (SBP) using inverse-variance weighted fixed-effects models in METAL [42]. The first GWAS was a meta-analysis of ≤757,601 individuals of European descent in the UK Biobank and International Consortium of Blood Pressure-Genome Wide Association Studies (ICBP) [43]. The second GWAS was performed in 99,785 individuals in the Genetic Epidemiology Research on Adult Health and Aging (GERA) cohort, of whom the majority (81.0%) were of European ancestry [44]. Both GWAS were adjusted for age, sex, body mass index (BMI), and antihypertensive medication use. Estimates that were genome-wide significant (*P* < 5.0 × 10^−8^) in pooled analyses (*N* ≤ 857,386) and that showed concordant direction of effect across both GWAS were then used to generate instruments.

To proxy ADRB1 inhibition, 8 single-nucleotide polymorphisms (SNPs) associated with SBP at genome-wide significance and within ±100 kb windows from *ADRB1* were obtained. To proxy NCC inhibition, 1 SNP associated with SBP at genome-wide significance and within a ±100-kb window from *SLC12A3* (alias for *NCC*) was obtained. For both of these drug targets, SNPs used as proxies were permitted to be in weak linkage disequilibrium (r^2^ < 0.10) with each other to increase the proportion of variance in each respective drug target explained by the instrument, maximizing instrument strength.

Since pooled GWAS estimates were obtained from analyses adjusted for BMI, which could induce collider bias, we also examined constructing instruments using summary genetic association data from a previous GWAS of SBP in 340,159 individuals in UK Biobank without adjustment for BMI or antihypertensive medication use (**S1 Table**) [45].

We explored construction of genetic instruments to proxy ACE inhibition using 2 approaches: (i) by obtaining genome-wide significant variants in weak linkage disequilibrium (r^2^ < 0.10) in or within ±100 kb from *ACE* that were associated with SBP in previously described pooled GWAS analyses (resulting in 2 SNPs); and (ii) by obtaining genome-wide significant variants in weak linkage disequilibrium (r^2^ < 0.10) in or within ±100 kb from *ACE* that were associated with serum ACE concentrations in a GWAS of 4,174 participants in the Outcome Reduction with Initial Glargine INtervention (ORIGIN) study (resulting in 14 SNPs) [46]. Approximately 46.6% of participants in the ORIGIN study were of European ancestry, and 53.4% were of Latin American ancestry. Effect allele frequencies for these 14 SNPs were broadly similar across both ancestries (**S2 Table**). We then compared the proportion of variance in either SBP or serum ACE concentrations explained (r^2^) across each respective instrument to prioritize the primary instrument to proxy ACE inhibition. The “serum ACE concentrations instrument” (r^2^ = 0.34 to 0.39, F = 2,156.5 to 2,594.9) was prioritized as our primary instrument to examine genetically proxied ACE inhibition because of stronger instrument strength as compared to the “SBP instrument” (r^2^ = 0.02, F = 128.5). In sensitivity analyses, we also examined the association between genetically proxied ACE inhibition and cancer endpoints using the “SBP instrument.”

As an additional instrument construction step, we also performed a post hoc comparison of the proportion of variance in serum ACE concentrations explained by both instruments and found that the serum ACE concentrations instrument explained a larger proportion of the variance in this trait than the SBP instrument (r^2^ = 0.28, F = 759.9).

To validate the serum ACE concentrations instrument, we examined the association between genetically proxied ACE inhibition and (i) SBP; (ii) risk of stroke in the MEGASTROKE consortium (40,585 cases; 406,111 controls of European ancestry); (iii) risk of coronary artery disease in the CARDIoGRAMplusC4D consortium (60,801 cases; 123,504 controls, 77% of whom were of European ancestry); and (iv) risk of type 2 diabetes in the DIAGRAM consortium (*N =* 74,124 cases; 824,006 controls of European ancestry) and compared the direction of effect estimates obtained with those reported for ACE inhibitor use in meta-analyses of randomized controlled trials [47–49]. Likewise, we validated ADRB1 and NCC instruments by examining the association between inhibition of these targets and risk of stroke and coronary artery disease, as reported in meta-analyses of clinical trials [49].

For analyses in individuals of East Asian ancestry, 1 *cis*-acting variant (rs4343) associated with ACE activity (*P* = 3.0 × 10^−25^) in a GWAS of 623 individuals with young onset hypertension of Han Chinese descent was obtained [50]. In the Japanese Biobank (*N =* 136,597), the A allele of rs4343 has previously been shown to associate with lower SBP (−0.26 mm Hg SBP, 95% CI −0.11 to −0.42; *P* = 6.7 × 10^−4^) [51]. This variant explained 0.008% of the variance of SBP (F = 11.6).

### Mendelian randomization primary and sensitivity analyses

Inverse-variance weighted random-effects models (permitting heterogeneity in causal estimates) were employed to estimate causal effects of genetically proxied drug target inhibition on cancer risk [52]. These models were adjusted for weak linkage disequilibrium between SNPs (r^2^ < 0.10) with reference to the 1,000 Genomes Phase 3 reference panel [53,54]. If underdispersion in causal estimates generated from individual genetic variants was present, the residual standard error was set to 1.

Mendelian randomization analysis assumes that the genetic instrument used to proxy a drug target (i) is associated with the drug target (“relevance”); (ii) does not share a common cause with the outcome (“exchangeability”); and (iii) affects the outcome only through the drug target (“exclusion restriction”).

We tested the “relevance” assumption by generating estimates of the proportion of variance of each drug target explained by the instrument (r^2^) and F-statistics. F-statistics can be used to examine whether results are likely to be influenced by weak instrument bias, i.e., reduced statistical power when an instrument explains a limited proportion of the variance in a drug target. As a convention, an F-statistic of at least 10 is indicative of minimal weak instrument bias [55].

We evaluated the “exclusion restriction” assumption by performing various sensitivity analyses. First, we performed colocalization to examine whether drug targets and cancer endpoints showing nominal evidence of an association in MR analyses (*P* < 0.05) share the same causal variant at a given locus. Such an analysis can permit exploration of whether drug targets and cancer outcomes are influenced by distinct causal variants that are in linkage disequilibrium with each other, indicative of horizontal pleiotropy (an instrument influencing an outcome through pathways independent to that of the exposure), a violation of the exclusion restriction criterion [56]. Colocalization analysis was performed by generating ±300 kb windows from the top SNP used to proxy each respective drug target. As a convention, a posterior probability of ≥0.80 was used to indicate support for a configuration tested. An extended description of colocalization analysis including assumptions of this method is presented in **S1 Methods**.

For analyses showing evidence of colocalization across drug target and cancer endpoint signals, we then examined whether there was evidence of an association of genetically proxied inhibition of that target with previously reported risk factors for the relevant cancer endpoint (i.e., BMI, low-density lipoprotein cholesterol, total cholesterol, iron, insulin-like growth factor 1, alcohol intake, standing height, and physical activity for colorectal cancer risk) [57–65]. If there was evidence for an association between a genetically proxied drug target and previously reported risk factor (*P* < 0.05), this could reflect vertical pleiotropy (i.e. “mediated pleiotropy” where an instrument has an effect on 2 or more traits that influence an outcome via the same biological pathway) or horizontal pleiotropy. In the presence of an association with a previously reported risk factor, multivariable mendelian randomization can then be used to examine the association of drug target inhibition in relation to cancer risk, accounting for this risk factor [45].

As an additional post hoc sensitivity analysis, we also evaluated whether SNPs used to instrument ADRB1 and NCC inhibition were also expression quantitative trait loci (eQTLs) for the genes encoding these proteins. Instrument validation and cancer endpoint mendelian randomization analyses were then repeated by restricting instruments to SNPs showing evidence of being eQTLs for these targets (Additional information on these sensitivity analyses is provided in **S2 Methods**).

Finally, iterative leave-one-out analysis was performed iteratively removing 1 SNP at a time from instruments to examine whether findings were driven by a single influential SNP.

To account for multiple testing across primary drug target analyses, a Bonferroni correction was used to establish a *P* value threshold of <0.0014 (false positive rate = 0.05/36 statistical tests [3 drug targets tested against 12 cancer endpoints]), which we used as a heuristic to define “strong evidence,” with findings between *P* ≥ 0.0014 and *P* < 0.20 defined as “weak evidence.”

### Colon transcriptome-wide GRS analysis

To explore potential mechanisms governing associations and to further evaluate potential violations of mendelian randomization assumptions, we examined associations between a genetic risk score for serum ACE concentrations and gene expression profiles in normal (i.e., nonneoplastic) colon tissue samples. Gene expression analysis was performed using data from the University of Barcelona and the University of Virginia Genotyping and RNA Sequencing Project (BarcUVa-Seq) [66]. This analysis was restricted to 445 individuals (mean age 60 years, 64% female, 95% of European ancestry) who participated in a Spanish colorectal cancer risk screening program that obtained a normal colonoscopy result (i.e., macroscopically normal colon tissue, with no malignant lesions). Further information on RNA-Seq data processing and quality control is presented in **S3 Methods**.

To perform transcriptome-wide analyses, weighted genetic risk scores (wGRS) to proxy serum ACE concentrations were constructed using 14 ACE SNPs in Plink v1.9 [67]. Expression levels for 21,482 genes (expressed as inverse normal transformed trimmed mean of M-values) were regressed on the standardized wGRS and adjusted for sex, the top 2 principal components of genetic ancestry, sequencing batch, probabilistic estimation of expression residuals (PEER) factors, and colon anatomical location. To account for multiple testing, a Bonferroni correction was used to establish a *P* value threshold of <2.33 × 10^−6^ (false positive rate = 0.05/21,482 statistical tests).

Bioinformatic follow-up of findings from transcriptome-wide analysis was performed to further interrogate downstream perturbations of the ACE wGRS on gene expression profiles using gene set enrichment analysis and coexpression network analysis. In brief, these methods can either evaluate whether expression levels of genes associated with the ACE wGRS are enriched in relation to an a priori defined set of genes based on curated functional annotation (gene set enrichment analysis) or permit the identification of clusters of genes (termed “modules” and assigned arbitrary color codes), which show a coordinated expression pattern associated with the wGRS (coexpression network analysis). Further information on gene set enrichment and coexpression network analysis is presented in **S4 Methods**.

There was no formal prespecified protocol for this study. All analyses described above were decided a priori except those designated as “post hoc” where additional sensitivity analyses were performed in response to peer review comments. This study is reported as per the Guidelines for strengthening the reporting of mendelian randomization studies (STROBE-MR) checklist (**S1 STROBE Checklist**) [68]. All statistical analyses were performed using R version 3.3.1.

## Results

Across the 3 drug targets that we examined, conservative estimates of F-statistics for their respective genetic instruments ranged from 269.1 to 2,156.5, suggesting that our analyses were unlikely to suffer from weak instrument bias. Characteristics of genetic variants in *ACE*, *ADRB1*, and *SLC12A3* used to proxy each pharmacological target are presented in **Table 1**. Estimates of r^2^ and F-statistics for each target are presented in **S3 Table**.

**Table 1:** Characteristics of SBP lowering genetic variants in *ACE*, *ADRB1*, and *SLC12A3*.

Target	Effect allele/Noneffect Allele	Effect Allele Frequency	Effect (SE)	*P* value
*ACE*				
rs4343	A/G	0.45	−0.63 (0.02)	1.53 × 10^−213^
rs12452187	A/G	0.60	−0.23 (0.02)	2.53 × 10^−27^
rs79480822	C/T	0.93	−0.55 (0.05)	6.37 × 10^−24^
rs3730025	G/A	0.01	−0.80 (0.09)	4.32 × 10^−19^
rs11655956	C/G	0.08	−0.35 (0.04)	1.06 × 10^−15^
rs118121655	G/A	0.96	−0.54 (0.07)	3.10 × 10^−15^
rs4365	G/A	0.97	−0.58 (0.08)	7.06 × 10^−12^
rs4968771	G/A	0.08	−0.22 (0.03)	1.78 × 10^−11^
rs12150648	G/A	0.96	−0.39 (0.06)	1.88 × 10^−10^
rs80311894	T/G	0.97	−0.46 (0.07)	2.60 × 10^−10^
rs118138685	C/G	0.04	−0.40 (0.07)	2.44 × 10^−9^
rs13342595	C/T	0.23	−0.14 (0.02)	2.48 × 10^−9^
rs28656895	T/C	0.23	−0.14 (0.02)	3.77 × 10^−9^
rs4968780	C/A	0.05	−0.28 (0.05)	1.86 × 10^−8^
*ADRB1*				
rs1801253	G/C	0.23	−0.41 (0.03)	8.07 × 10^−43^
rs11196549	G/A	0.96	−0.62 (0.07)	2.53 × 10^−19^
rs4918889	G/C	0.17	−0.30 (0.04)	7.53 × 10^−18^
rs460718	A/G	0.33	−0.24 (0.03)	2.21 × 10^−17^
rs11196597	G/A	0.86	−0.27 (0.04)	3.07 × 10^−12^
rs143854972	G/A	0.94	−0.39 (0.06)	4.35 × 10^−11^
rs17875473	C/T	0.91	−0.28 (0.05)	9.04 × 10^−9^
rs10787510	A/G	0.48	−0.15 (0.03)	2.01 × 10^−8^
*NCC*				
rs35797045	A/C	0.05	−0.35 (0.06)	4.85 × 10^−8^

Effect (SE) represents change in serum ACE concentrations per additional copy of the effect allele for ACE analysis and change in SBP per additional copy of the effect allele for ADRB1 and NCC analyses. In analyses of genetically proxied ACE inhibition and colorectal cancer risk, 1 SNP (rs8064760) was not available in the colorectal cancer dataset. Two SNPs associated with SBP used to proxy ACE inhibition in sensitivity analyses were as follows: rs8077276 (effect allele/noneffect allele: A/G, effect (se): −0.27 (0.03), effect allele frequency: 0.62; *P* value: 4.47 × 10^−22^) and rs28656895 (effect allele/noneffect allele: T/C, effect (se): −0.19 (0.03), effect allele frequency: 0.23; *P* value: 3.37 × 10^−9^).

ACE, angiotensin-converting enzyme; ADRB1, β-1 adrenergic receptor; NCC, sodium-chloride symporter; SBP, systolic blood pressure; SNP, single-nucleotide polymorphism.

### Instrument validation

Findings from genetic instrument validation analyses for drug targets were broadly concordant (i.e., in direction of effect) with findings from meta-analyses of randomized trials for these medications. Genetically proxied ACE inhibition was associated with lower SBP (mm Hg per SD lower serum ACE concentration: −0.40, 95% CI −0.21 to −0.59, *P* = 4.2 × 10^−5^) and a lower risk of type 2 diabetes (odds ratio (OR) equivalent to 1 mm Hg lower SBP: 0.90, 95% CI 0.85 to 0.95, *P* = 1.3 × 10^−4^). There was weak evidence for an association of genetically proxied ACE inhibition with lower risk of stroke (OR 0.94, 95% CI 0.88 to 1.01; *P* = 0.06) and coronary artery disease (OR 0.95, 95% CI 0.89 to 1.02; *P* = 0.16).

Genetically proxied ADRB1 inhibition was associated with lower risk of coronary artery disease (per 1 mm Hg lower SBP: OR 0.95, 95% CI 0.92 to 0.98; *P* = 1.5 × 10^−3^) and weakly associated with risk of stroke (OR 1.03, 95% CI 0.99 to 1.07; *P* = 0.18).

Genetically proxied NCC inhibition was associated with lower risk of coronary artery disease (per 1 mm Hg lower SBP: OR 0.81, 95% CI 0.81, 95% CI 0.71 to 0.93, *P* = 3.2 × 10^−3^) and was weakly associated with lower risk of stroke (OR 0.89, 95% CI 0.78 to 1.02; *P* = 0.10).

### Genetically proxied ACE inhibition and cancer risk

Genetically proxied ACE inhibition was associated with an increased odds of colorectal cancer (OR equivalent to 1 mm Hg lower SBP: 1.13, 95% CI 1.06 to 1.22; *P* = 3.6 × 10^−4^). Likewise, in analyses using SBP SNPs in *ACE*, genetically proxied SBP lowering via ACE inhibition was associated with an increased odds of colorectal cancer (OR equivalent to 1 mm Hg lower SBP: 1.11, 95% CI 1.04 to 1.18; *P* = 1.3 × 10^−3^). When scaled to represent SBP lowering achieved in clinical trials of ACE inhibitors for primary hypertension (equivalent to 8 mm Hg lower SBP), this represents an OR of 2.74 (95% CI 1.58 to 4.76) [69]. In site-specific analyses, this association was stronger for colon cancer risk (OR 1.18, 95% CI 1.07 to 1.31; *P* = 9.7 × 10^−4^) than rectal cancer risk (OR 1.07, 95% CI 0.97 to 1.18; *P* = 0.16). Similar associations were found across risk of proximal colon cancer (OR 1.23, 95% CI 1.10 to 1.37; *P* = 1.9 × 10^−4^) and distal colon cancer (OR 1.15, 95% CI 1.03 to 1.27; *P* = 0.01).

**Fig 1:**
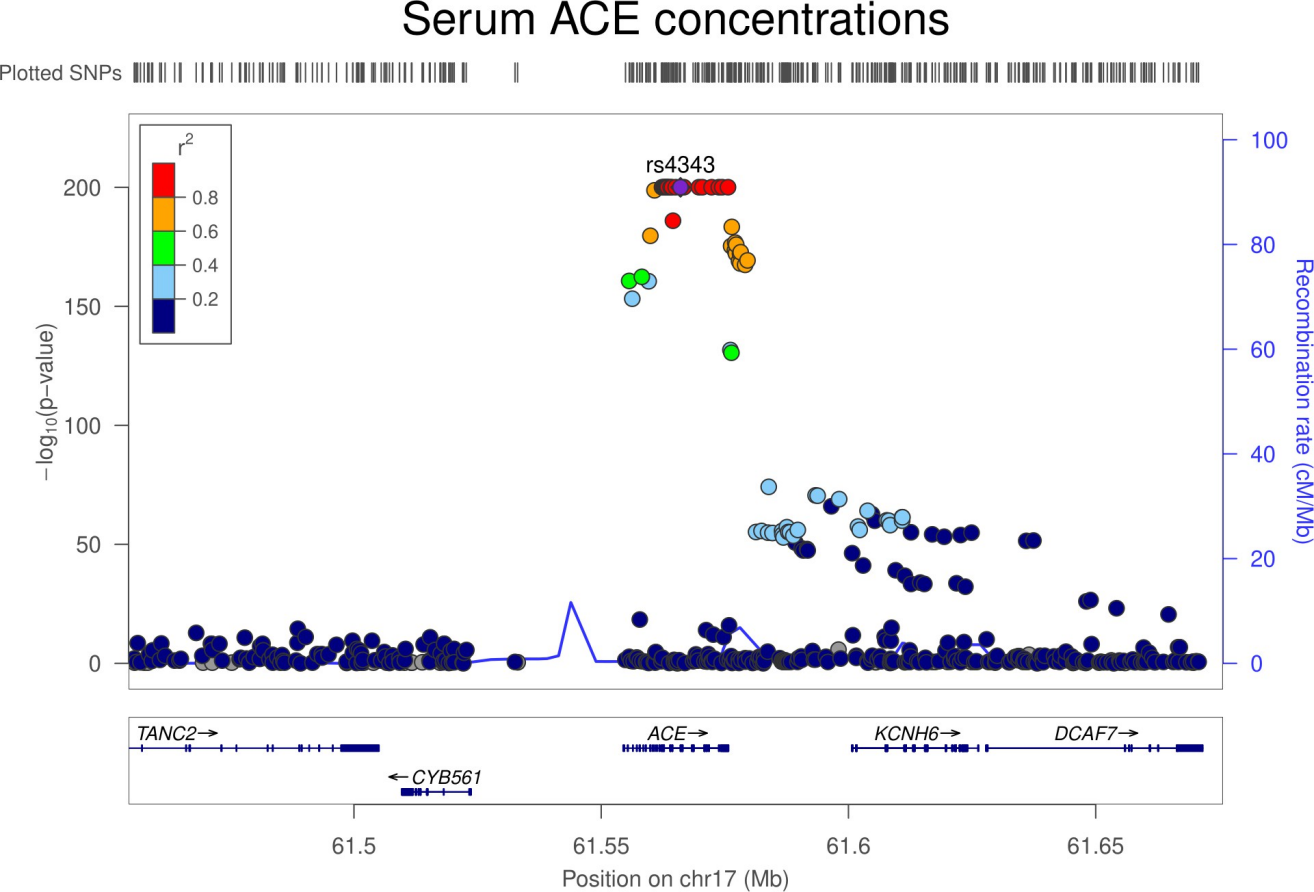
Regional Manhattan plot of associations of SNPs with serum ACE concentrations ±300 kb from the SNP used to proxy serum ACE concentrations (rs4343) in the *ACE* region. ACE, angiotensin-converting enzyme; Mb, Megabase; SNP, single-nucleotide polymorphism.

**Fig 2:**
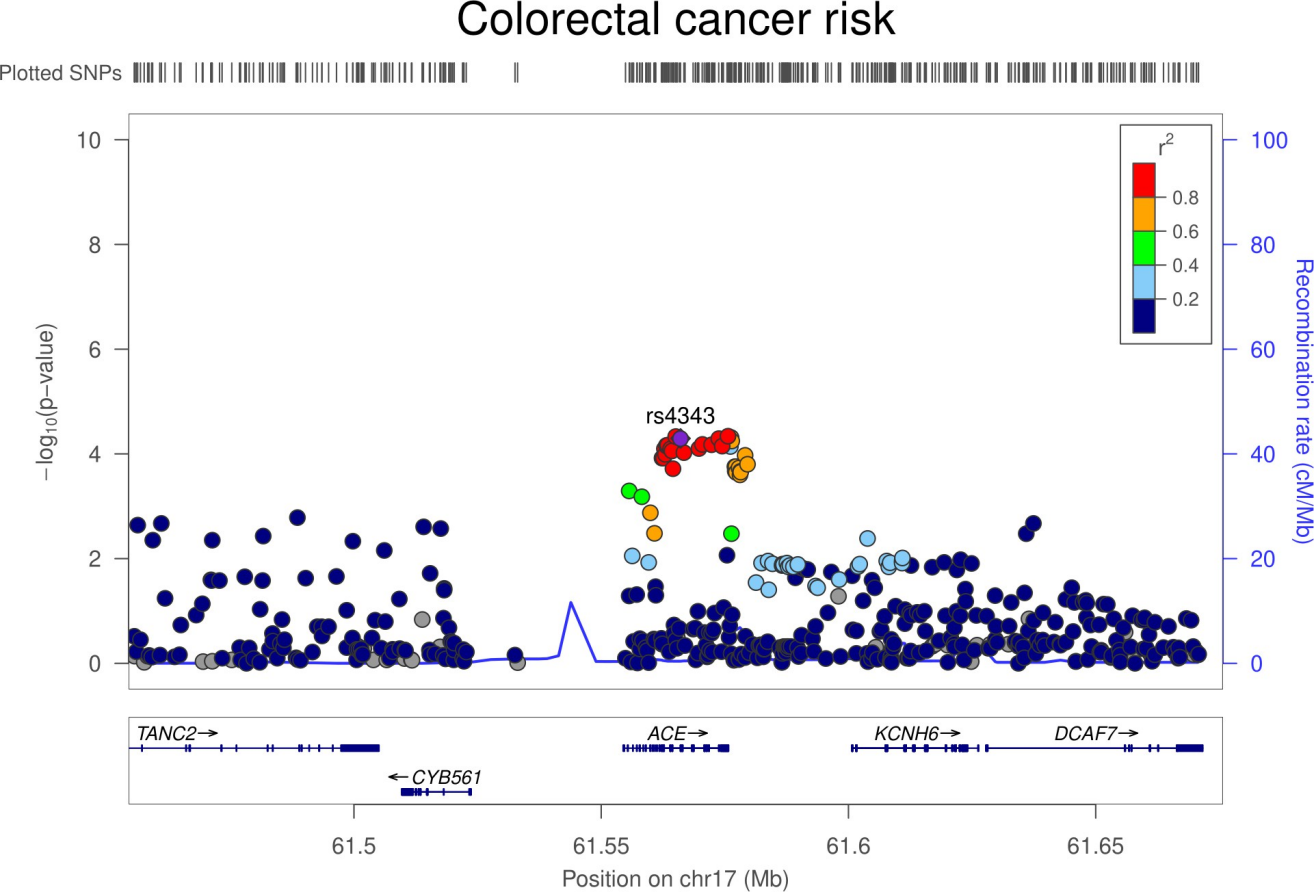
Regional Manhattan plot of associations of SNPs with colorectal cancer risk ±300 kb from the SNP used to proxy serum ACE concentrations (rs4343) in the *ACE* region. ACE, angiotensin-converting enzyme; Mb, Megabase; SNP, single-nucleotide polymorphism.

Colocalization analysis suggested that serum ACE and colorectal cancer associations had a 91.4% posterior probability of sharing a causal variant within the *ACE* locus (**S4 Table**). Regional Manhattan plots examining the association of all SNPs ±300 kb from the top SNP for serum ACE concentrations (rs4343) for their association with serum ACE concentrations (**Fig 1**) and with colorectal cancer risk (**Fig 2**) did not appear to support the presence of 2 or more independent causal variants driving associations across either trait.

In mendelian randomization analyses examining the association of genetically proxied ACE inhibition with 8 previously reported colorectal cancer risk factors, there was little evidence to support associations (**S5 Table**). There was also little evidence to support an association of genetically proxied SBP with colorectal cancer risk (OR per 1 mmHg lower SBP: 1.00, 95% CI 0.99 to 1.01; *P* = 0.50), suggesting a potential mechanism-specific effect of this drug target on colorectal cancer risk.

Additionally, results of analyses that iteratively removed one SNP at a time from the instrument and recalculated the overall mendelian randomization estimate were consistent, suggesting that associations were not being driven through individual influential SNPs (**S6 Table**).

There was little evidence that genetically proxied ACE inhibition was associated with risk of breast cancer (OR 0.98, 95% CI 0.94 to 1.02; *P* = 0.35) or lung cancer (OR 1.01, 95% CI 0.92 to 1.10; *P* = 0.93) and weak evidence for an association with prostate cancer risk (OR 1.06, 95% CI 0.99 to 1.13; *P* = 0.08). Likewise, there was little evidence of association of genetically proxied ACE inhibition with these cancers in histological subtype-stratified analyses (**Table 2**).

**Table 2:** Association between genetically proxied ACE inhibition and risk of overall and subtype-specific breast, colorectal, prostate, and lung cancer risk.

Outcome	N (cases, controls)	OR (95% CI)	*P* value
Breast cancer	122,977; 105,974	0.98 (0.94–1.02)	0.35
ER+ Breast cancer	69,501; 105,974	0.99 (0.94–1.04)	0.76
ER− Breast cancer	21,468; 105,974	0.97 (0.90–1.05)	0.47
Colorectal cancer	58,221; 67,694	1.13 (1.06–1.22)	3.6 × 10^−4^
Colon cancer	32,002; 64,159	1.18 (1.07–1.31)	9.7 × 10^−4^
Rectal cancer	16,212; 64,159	1.07 (0.97–1.18)	0.16
Lung cancer	29,863; 55,586	1.01 (0.92–1.10)	0.93
Lung adenocarcinoma	11,245; 54,619	1.02 (0.91–1.15)	0.70
Small cell lung carcinoma	2,791; 20,580	0.96 (0.76–1.20)	0.71
Squamous cell lung cancer	7,704; 54,763	0.97 (0.81–1.16)	0.73
Prostate cancer	79,148; 61,106	1.06 (0.99–1.13)	0.08
Advanced prostate cancer	15,167; 58,308	1.05 (0.94–1.17)	0.37

ACE, angiotensin-converting enzyme; CI, confidence interval; ER, estrogen receptor; OR, odds ratio, SBP, systolic blood pressure.

OR represents the exponential change in odds of cancer per genetically proxied inhibition of ACE equivalent to a 1-mm Hg decrease in SBP.

### Genetically proxied ADRB1 inhibition and cancer risk

**Fig 3:**
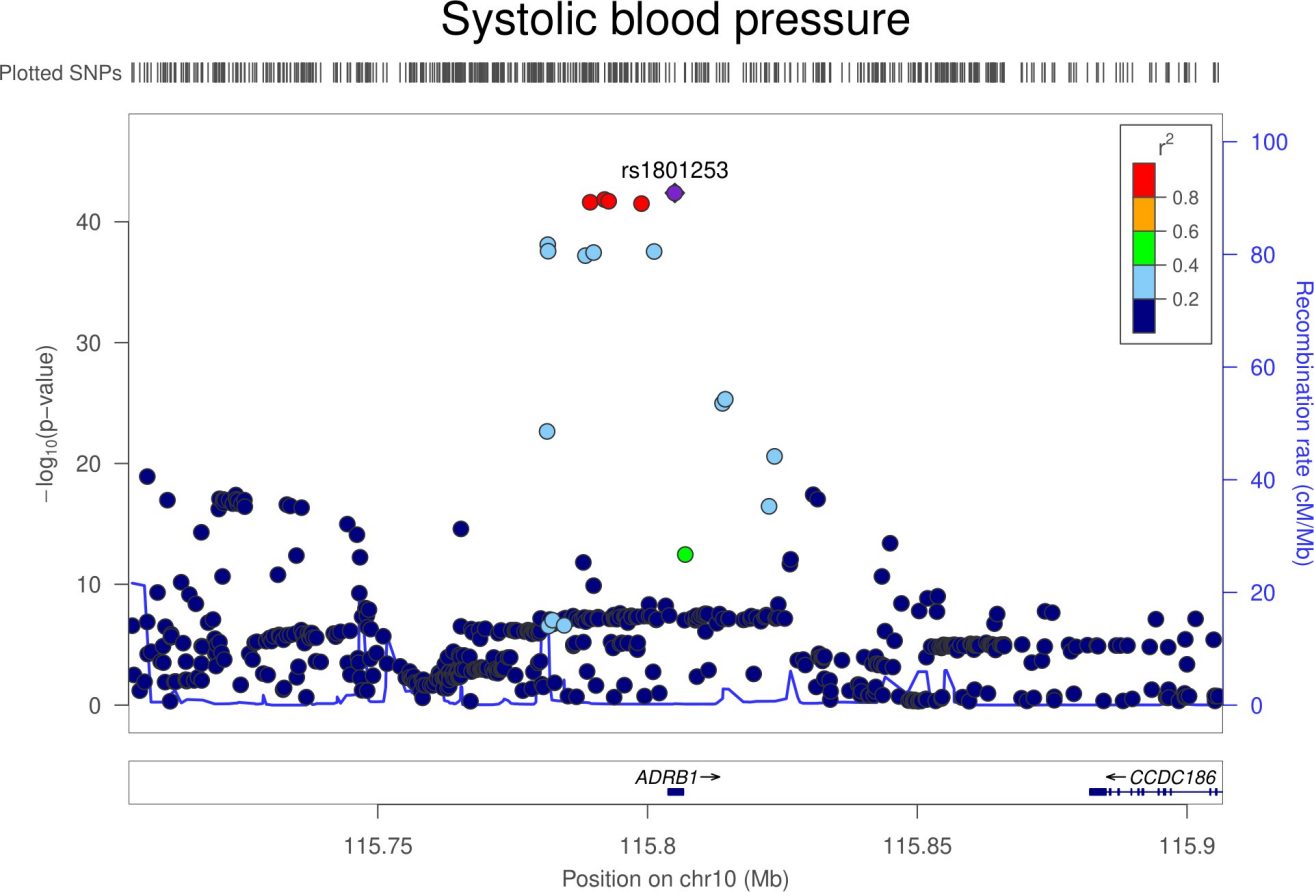
Regional Manhattan plot of associations of SNPs with SBP ±300 kb from the SNP used to proxy SBP (rs1801253) in the *ADRB1* region. Mb, Megabase; SBP, systolic blood pressure; SNP, single-nucleotide polymorphism.

**Fig 4:**
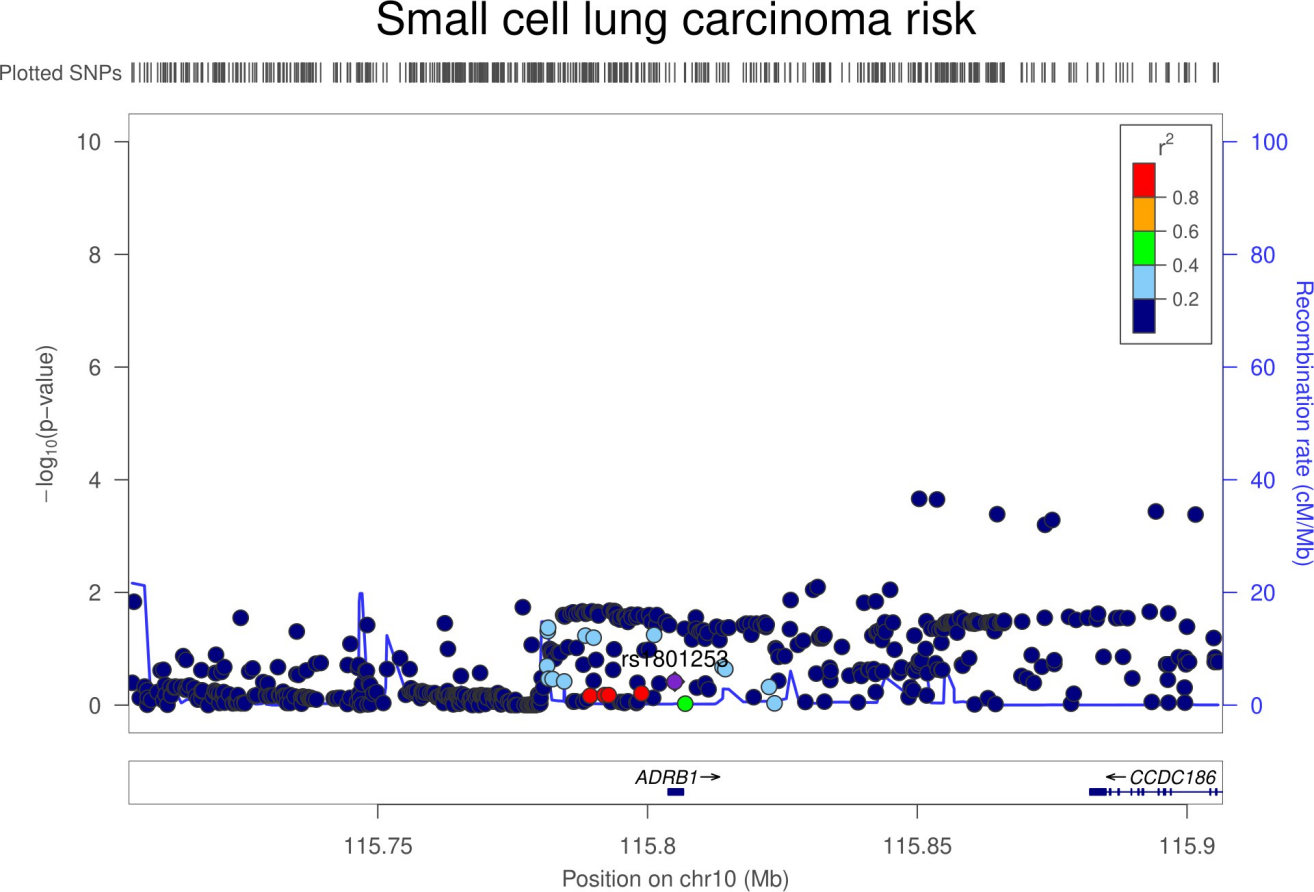
Regional Manhattan plot of associations of SNPs with small cell lung carcinoma risk ±300 kb from the SNP used to proxy SBP (rs1801253) in the *ADRB1* region. Mb, Megabase; SBP, systolic blood pressure; SNP, single-nucleotide polymorphism.

There was little evidence that genetically proxied ADRB1 inhibition was associated with overall risk of breast, colorectal, lung, or prostate cancer (**Table 3**). In lung cancer subtype-stratified analyses, there was weak evidence to suggest an association of genetically proxied ADRB1 inhibition with lower risk of small cell lung carcinoma (OR equivalent to 1 mm Hg lower SBP: 0.87, 95% CI 0.79 to 0.96; *P* = 0.008). Colocalization analysis suggested that ADRB1 and small cell lung carcinoma were unlikely to share a causal variant within the *ADRB1* locus (1.5% posterior probability of a shared causal variant) (**S7 Table, Figs 3 and 4**). Findings for overall and subtype-specific cancer risk did not differ markedly when using an instrument for ADRB1 inhibition constructed from a GWAS unadjusted for BMI (**S8 Table**). In sensitivity analyses restricting the ADRB1 instrument to SNPs that are eQTLs for ADRB1, findings were consistent with those obtained when using the primary instrument for this target (**S10 Table**).

**Table 3:** Association between genetically proxied ADRB1 inhibition and risk of overall and subtype-specific breast, colorectal, prostate, and lung cancer risk.

Outcome	N (cases, controls)	OR (95% CI)	*P* value
Breast cancer	122,977; 105,974	1.01 (0.99–1.04)	0.38
ER+ Breast cancer	69,501; 105,974	1.01 (0.98–1.04)	0.44
ER− Breast cancer	21,468; 105,974	0.98 (0.94–1.02)	0.38
Colorectal cancer	58,221; 67,694	0.98 (0.96–1.01)	0.31
Colon cancer	32,002; 64,159	0.99 (0.95–1.03)	0.63
Rectal cancer	16,212; 64,159	1.00 (0.95–1.04)	0.84
Lung cancer	29,863; 55,586	1.01 (0.96–1.07)	0.64
Lung adenocarcinoma	11,245; 54,619	0.98 (0.91–1.04)	0.48
Small cell lung carcinoma	2,791; 20,580	0.87 (0.79–0.96)	0.008
Squamous cell lung cancer	7,704; 54,763	0.98 (0.91–1.06)	0.67
Prostate cancer	79,148; 61,106	1.00 (0.96–1.03)	0.73
Advanced prostate cancer	15,167; 58,308	1.00 (0.94–1.06)	0.97

ADRB1, β-1 adrenergic receptor; CI, confidence interval; ER, estrogen receptor; OR, odds ratio, SBP, systolic blood pressure.

OR represents the exponential change in odds of cancer per genetically proxied inhibition of ADRB1 equivalent to a 1-mm Hg decrease in SBP.

### Genetically proxied NCC inhibition and cancer risk

**Fig 5:**
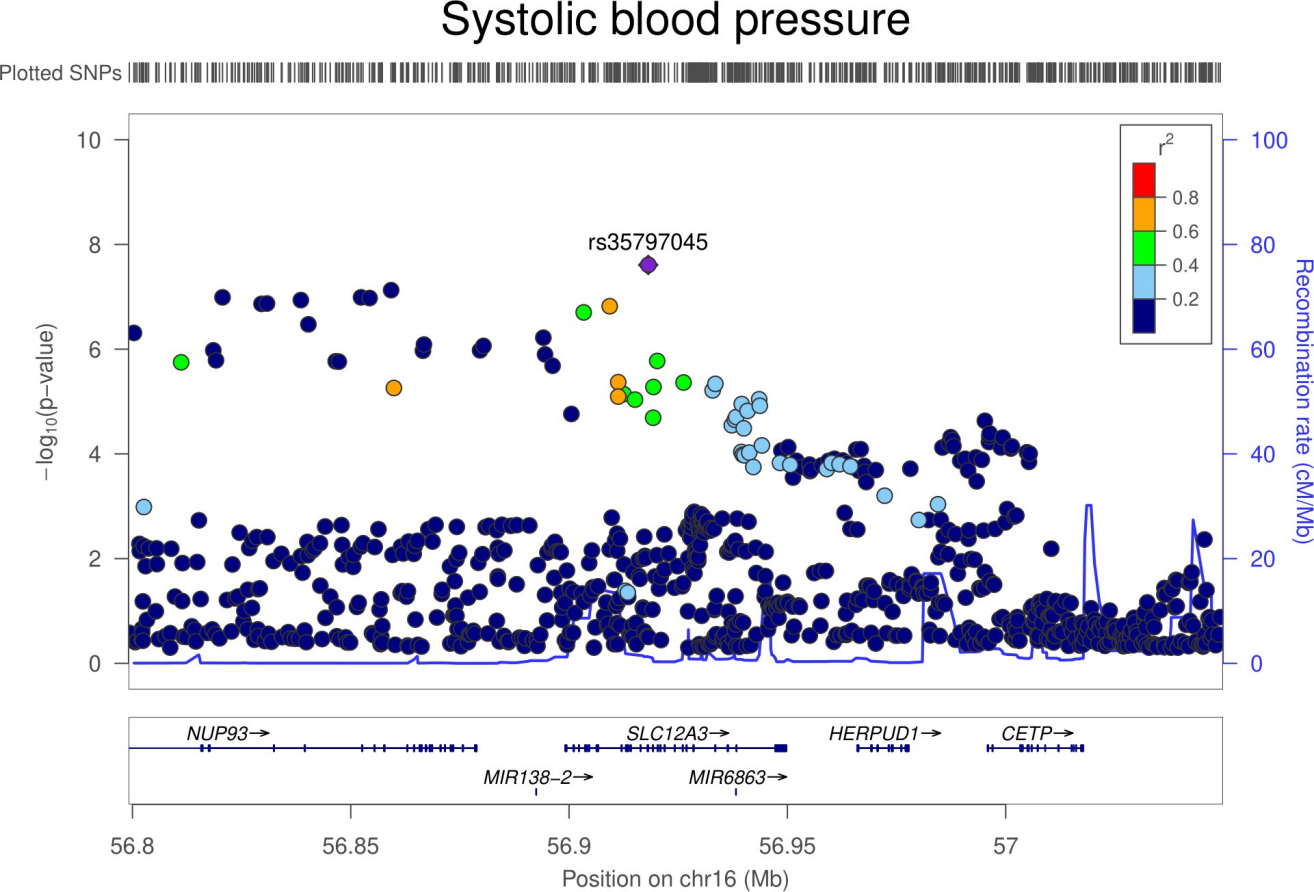
Regional Manhattan plot of associations of SNPs with SBP ±300 kb from the SNP used to proxy SBP (rs35797045) in the *SLC12A3* region. Mb, Megabase; SBP, systolic blood pressure; SNP, single-nucleotide polymorphism.

**Fig 6:**
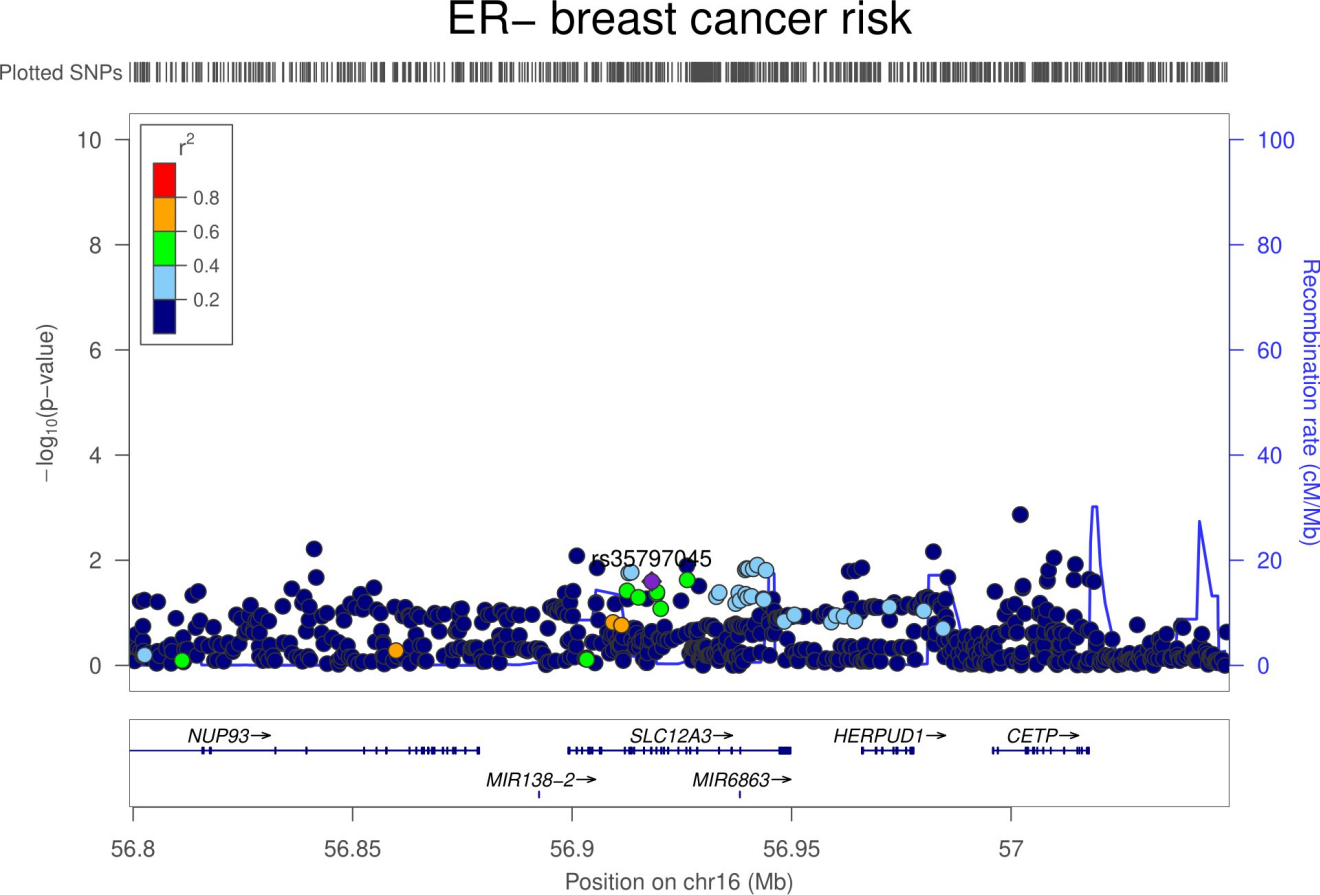
Regional Manhattan plot of associations of SNPs with ER− breast cancer risk ±300 kb from the SNP used to proxy SBP (rs35797045) in the *SLC12A3* region. ER, estrogen receptor; Mb, Megabase; SBP, systolic blood pressure; SNP, single-nucleotide polymorphism.

There was little evidence that genetically proxied NCC inhibition was associated with overall risk of breast, colorectal, lung, or prostate cancer (**Table 4**). In ER–stratified breast cancer analyses, there was weak evidence that NCC inhibition was associated with an increased risk of ER− breast cancer (OR equivalent to 1 mm Hg lower SBP: 1.20, 95% CI 1.02 to 1.40; *P* = 0.03). Colocalization analysis provided little support for NCC and ER− breast cancer association sharing a causal variant within the *SLC12A3* locus (11.5% posterior probability of a shared causal variant) (**S11 Table, Figs 5 and 6**).

**Table 4:** Association between genetically proxied NCC inhibition and risk of overall and subtype-specific breast, colorectal, prostate, and lung cancer risk.

Outcome	N (cases, controls)	OR (95% CI)	*P* value
Breast cancer	122,977; 105,974	1.08 (0.99–1.18)	0.08
ER+ Breast cancer	69,501; 105,974	1.06 (0.95–1.18)	0.28
ER− Breast cancer	21,468; 105,974	1.20 (1.02–1.40)	0.03
Colorectal cancer	58,221; 67,694	1.09 (0.96–1.23)	0.19
Colon cancer	32,002; 64,159	1.03 (0.89–1.19)	0.69
Rectal cancer	16,212; 64,159	1.13 (0.94–1.36)	0.20
Lung cancer	29,863; 55,586	1.09 (0.89–1.33)	0.38
Lung adenocarcinoma	11,245; 54,619	1.01 (0.81–1.26)	0.95
Small cell lung carcinoma	2,791; 20,580	1.12 (0.76–1.53)	0.57
Squamous cell lung cancer	7,704; 54,763	1.00 (0.78–1.29)	0.99
Prostate cancer	79,148; 61,106	1.08 (0.96–1.19)	0.18
Advanced prostate cancer	15,167; 58,308	1.05 (0.86–1.28)	0.63

CI, confidence interval; ER, estrogen receptor; NCC, sodium-chloride symporter; OR, odds ratio, SBP, systolic blood pressure.

OR represents the exponential change in odds of cancer per genetically proxied inhibition of NCC equivalent to a 1-mm Hg decrease in SBP.

### Replication analysis in Europeans and exploratory analysis in East Asians

Findings for genetically proxied ACE inhibition and colorectal cancer risk were replicated in an independent sample of 1,571 colorectal cancer cases and 120,006 controls of European ancestry in the Finngen consortium (1.40, 95% CI 1.02 to 1.92; *P* = 0.035). In analyses of 23,572 colorectal cancer cases and 48,700 controls of East Asian descent, there was little evidence of association of genetically proxied ACE inhibition and colorectal cancer risk (OR 0.97, 95% CI 0.88 to 1.07; *P* = 0.59).

### Colon gene expression analysis

**Fig 7:**
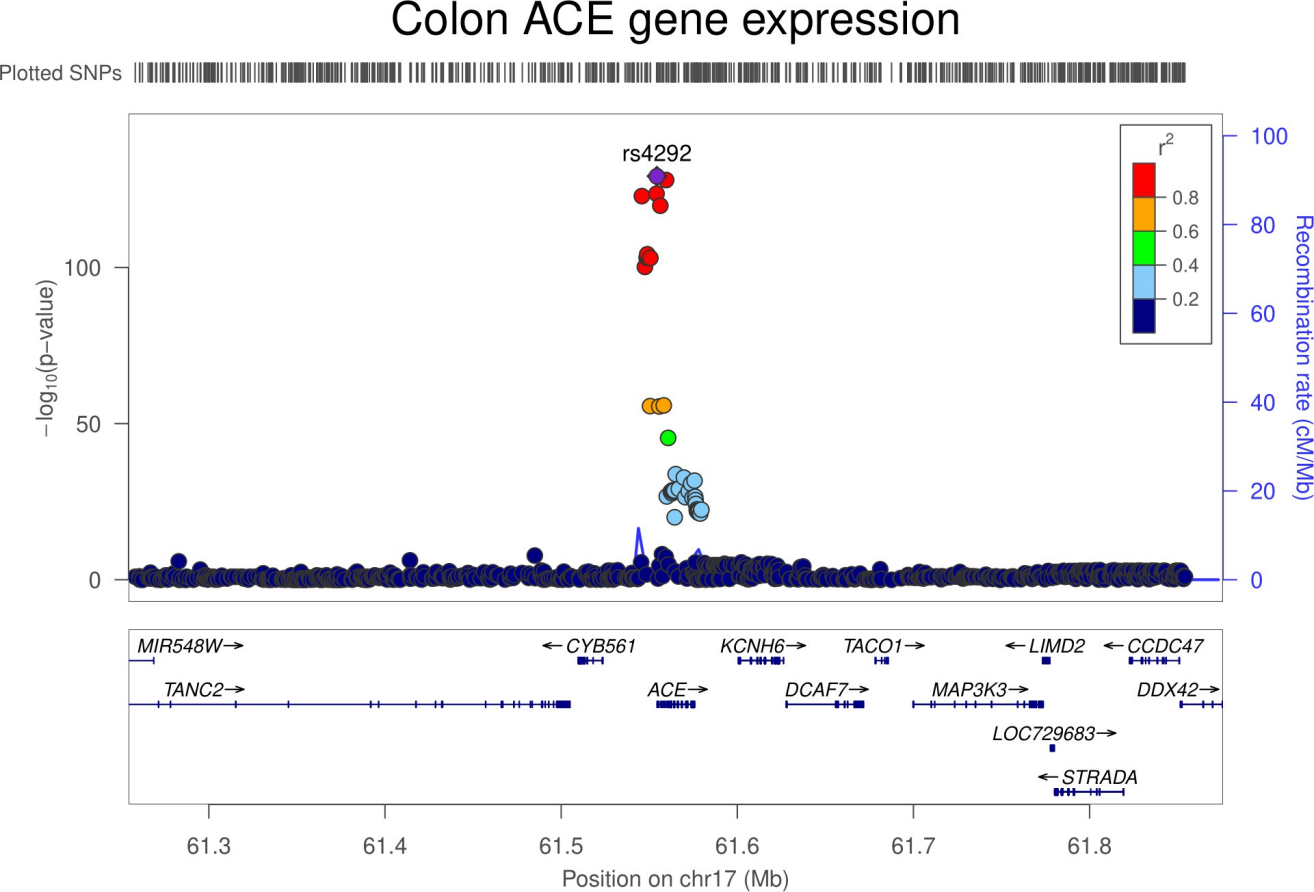
Regional Manhattan plot of associations of SNPs with colon ACE expression ±300 kb from the SNP used to proxy colon ACE expression (rs4292) in the *ACE* region. ACE, angiotensin-converting enzyme; Mb, Megabase; SNP, single-nucleotide polymorphism.

**Fig 8:**
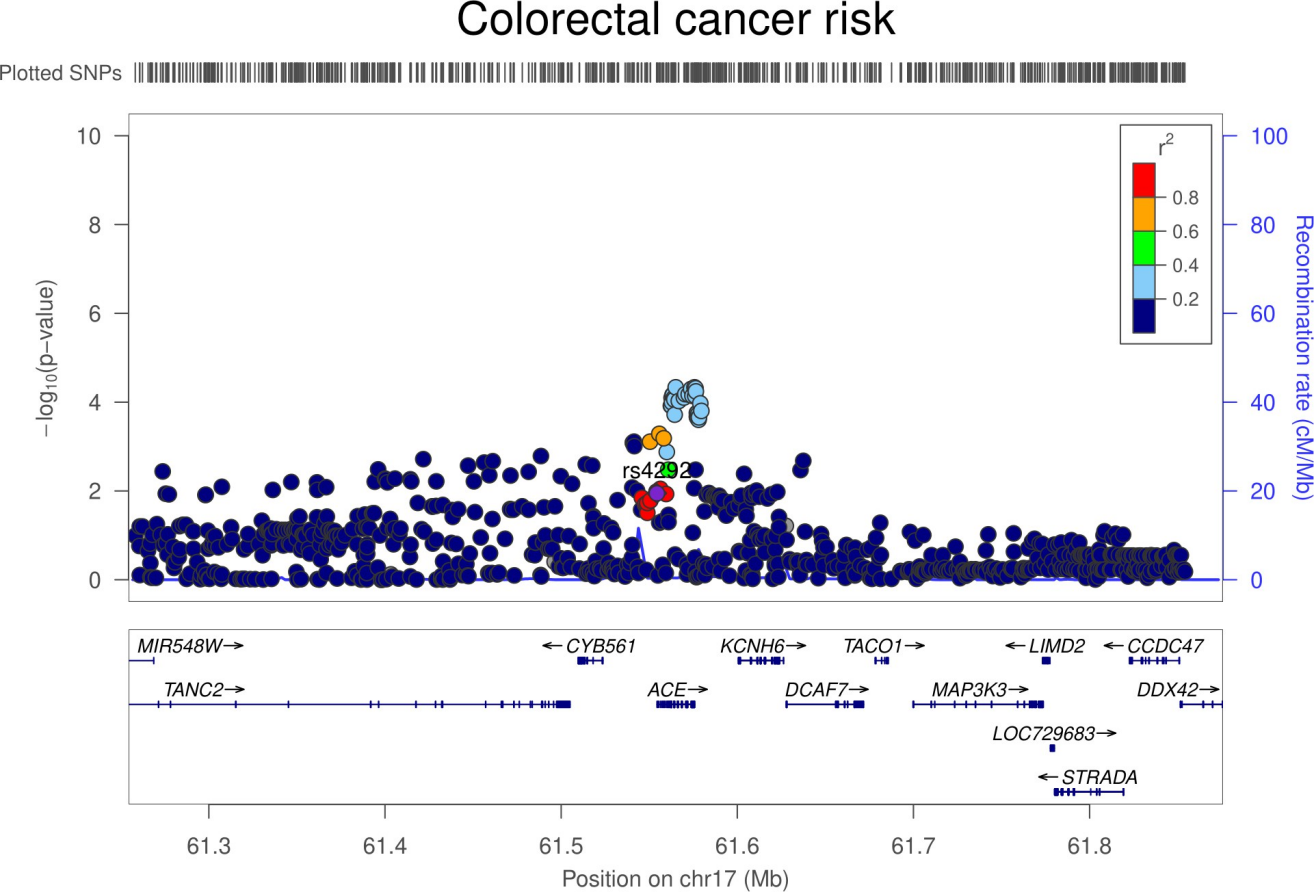
Regional Manhattan plot of associations of SNPs with colorectal cancer risk ±300 kb from the SNP used to proxy colon ACE expression (rs4292) in the *ACE* region. ACE, angiotensin-converting enzyme; Mb, Megabase; SNP, single-nucleotide polymorphism.

In transcriptome-wide analyses, the serum ACE wGRS was most strongly associated with *ACE* expression levels in the colon (trimmed mean of M-values [TMMs] per SD increase in wGRS: −0.42, 95% CI −0.49 to −0.36; *P* = 2.29 × 10^−31^). Genetically proxied *ACE* expression in the colon was associated with increased odds of colorectal cancer (OR per SD increase in expression: 1.02, 95% CI 1.00 to 1.04; *P* = 0.01). However, colocalization analysis suggested that colon *ACE* expression and colorectal cancer risk were unlikely to share a causal variant within the *ACE* locus (29.1% posterior probability of a shared causal variant) (**S12 Table, Figs 7 and 8**). The serum ACE wGRS was also associated with expression levels of C*YB561* (TMMs per SD increase in wGRS: −0.17, 95% CI −0.21 to −0.12; *P* = 8.28 × 10^−11^) and *FTSJ3* (TMMs per SD increase in wGRS: −0.19, 95% CI −0.24 to −0.13; *P* = 2.95 × 10^−10^) in the colon after correction for multiple testing. *ACE*, *CYB561*, and *FTSJ3* are neighboring genes on chromosome 17, suggesting that associations between the ACE wGRS and *CYB561* and *FTSJ3* could be driven through their coexpression. Genetically proxied *CYB561* expression in the colon was associated with increased odds of colorectal cancer (OR per SD increase in expression: 1.06, 95% CI 1.02 to 1.10; *P* = 0.005). However, multivariable mendelian randomization analysis examining the association of genetically proxied ACE inhibition with colorectal cancer risk adjusting for *CYB561* expression in the colon was consistent with univariable analyses (OR 1.13, 95% CI: 0.96 to 1.32; *P* = 0.14). Genetically proxied *FTSJ3* expression in the colon was not associated with odds of colorectal cancer (OR per SD increase in expression: 1.00, 95% CI 0.98 to 1.03; *P* = 0.77).

In gene set enrichment analysis of genes whose expression was associated with the serum ACE wGRS (*P* < 5.0 × 10^−3^), there was evidence for enrichment of expression of genes relating to memory CD8 T cells (as compared to effector CD8 T cells) in the immunologic signatures database (GSE10239) (*P* = 1.35 × 10^−6^) but little evidence for expression of other gene sets or pathways after correction for multiple testing.

In coexpression network analysis, 30 distinct modules were defined. *ACE* was in the black module along with another 659 genes. This module was correlated with the ACE wGRS (r = −0.11; *P* = 0.03). Gene set enrichment analysis of genes located in the black module showed evidence of enrichment in susceptibility genes for colorectal cancer (*P* = 1.00 × 10^−3^).

Complete findings from transcriptome-wide GRS and gene set enrichment analyses, along with genes from the black module from coexpression network analysis are presented in **S11–S14 Tables**.

## Discussion

In this mendelian randomization analysis of up to 289,612 cancer cases and 291,224 controls, genetically proxied long-term ACE inhibition was associated with an increased risk of colorectal cancer. This association was restricted to cancer of the colon, with similar associations across the proximal and distal colon. There was little evidence to support associations of genetically proxied ACE inhibition with risk of other cancers. Genetically proxied ADRB1 and NCC inhibition were not associated with risk of breast, colorectal, lung or prostate cancer.

Our findings for genetically proxied ACE inhibition and colorectal cancer risk are not consistent with some previous conventional observational analyses. A meta-analysis of 7 observational studies reported a protective association of ACE inhibitor use with colorectal cancer risk (OR 0.81 95% CI 0.70 to 0.92), though with substantial heterogeneity across studies (*I*^2^ = 71.1%) [14]. Interpretation of these findings is complicated by variable use of prevalent drug users, heterogenous comparator groups (both active controls and nondrug users), and the potential for immortal time bias across most included studies. Further, this meta-analysis did not include data from an earlier large Danish population-based case–control analysis with 15,560 colorectal cancer cases and 62,525 controls, which reported an increased risk of colorectal cancer (OR 1.30, 95% CI 1.22 to 1.39) among long-term users of ACE inhibitors (≥1,000 daily doses within 5 years of study entry), as compared to never-users [7].

The potential mechanisms underpinning an association between genetically proxied ACE inhibition and colorectal cancer risk are unclear. ACE is a multifaceted enzyme, capable of cleaving several different peptide substrates with potential roles in carcinogenesis [70]. Along with ACE inhibition leading to an accumulation of bradykinin and substance P, both potential inducers of tumor proliferation, ACE inhibition can also lead to an increase in Ac-SDKP, an endogenous antifibrotic peptide that is capable of inducing angiogenesis [71]. The observed restriction of an association of genetically proxied ACE inhibition with risk of colon, but not rectal, cancer is consistent with evidence that mRNA and protein levels of ACE are enriched in the colon but not in rectal tissue [72]. There was limited evidence of association of a serum ACE genetic risk score with distinct gene expression profiles in transcriptome-wide analyses. However, gene set enrichment analysis of these findings suggested enriched expression of genes involved in immunological pathways relating to memory CD8 T cells and coexpression network analysis identified ACE expression in a cluster of coexpressed genes enriched for colorectal cancer risk susceptibility genes (e.g., *LAMA5*, *PNKD*, *TOX2*, *PLEKHG6*) [73]. These findings suggest potential future avenues of exploration to uncover mechanistic pathways linking ACE with colorectal cancer risk.

Meta-analyses of randomized trials have not reported increased rates of cancer among ACE inhibitor users, though these analyses have not reported findings separately for colorectal cancer [2,3]. Potential discrepancies in findings for colorectal cancer between this mendelian randomization analysis and previous clinical trials could reflect the relatively short duration of these trials (median follow-up of 3.5 years) given long induction periods of colorectal cancer. For example, the “adenocarcinoma sequence” proposes that transformation of normal colorectal epithelium to an adenoma and ultimately to invasive and metastatic cancer may occur over the course of several decades [74,75]. Consistent with this long induction period, in randomized controlled trials examining the chemopreventive effect of aspirin on colorectal cancer risk, protective effects of aspirin are not seen until 7 years after initiation of treatment, with clear risk reductions becoming apparent only after 10 years of follow-up [76]. It is therefore possible that adverse effects of ACE inhibition on colorectal cancer risk may likewise not emerge until many years after treatment initiation. Alternatively, it may be possible that an effect of ACE inhibition on cancer is restricted solely to the earliest stages of the adenoma-carcinoma sequence and therefore may not influence cancer risk among largely middle-aged participants of clinical trials if dysplasia is already present. Finally, it is possible that lower levels of circulating ACE concentrations may influence colorectal cancer risk only during a particular critical or sensitive period of the life course (e.g., in childhood or adolescence), given some evidence to suggest a potential role of early-life factors in colorectal carcinogenesis [77].

Our largely null findings for genetically proxied ACE inhibition and risk of breast, lung, and prostate cancer risk are not consistent with some previous observational reports that compared ACE inhibitor users to nonusers or to users of β blockers or thiazide diuretics [7,9,17]. However, our findings for genetically proxied ACE inhibition are in agreement with those from short-term randomized controlled trials for these site-specific cancers and suggest that long-term use of these drugs may not influence cancer risk, though we cannot rule out small effects from their long-term use [3]. Likewise, our findings for ADRB1 and NCC are in agreement with short-term trial data reporting no association of β blockers and thiazide diuretics use with overall cancer risk [2].

Strengths of this analysis include the use of *cis-*acting variants in genes encoding antihypertensive drug targets to proxy inhibition of these targets, which should minimize confounding, the employment of various sensitivity analyses to rigorously assess for violations of mendelian randomization assumptions, and the use of a summary-data mendelian randomization approach, which permitted us to leverage large-scale genetic data from several cancer GWAS consortia, enhancing statistical power and precision of causal estimates. As with prior mendelian randomization analyses of antihypertensive drug targets that used similar approaches to instrument construction to our analysis, the general concordance of estimates of the effect of these instruments on cardiometabolic endpoints with those reported in prior clinical trials for these medications supports the plausibility of these instruments [78,79]. Finally, the use of germline genetic variants as proxies for antihypertensive drug targets facilitated evaluation of the effect of the long-term inhibition of these targets, which may be more representative of the typically decades-long use of antihypertensive therapy as compared to periods of medication use typically examined in conventional observational studies and randomized trials. Despite the evidence suggesting a link between genetically proxied ACE inhibition and colorectal cancer, however, these findings cannot demonstrate a causal relationship between ACE inhibitor use and colorectal cancer risk; only a randomized controlled trial could establish this relationship.

There are several limitations to these analyses. First, mendelian randomization analyses are restricted to examining on-target (i.e., target-mediated) effects of therapeutic interventions. Second, statistical power was likely limited in some analyses of less common cancer subtypes. Limited statistical power in analyses of genetically proxied ACE inhibition and colorectal cancer risk in East Asians (instrumented by rs4343) may also have accounted for the lack of association between these traits within this population. Identification of stronger genetic instruments for ACE inhibition in East Asian populations can help to uncover whether the lack of transportability of ACE and colorectal cancer findings from Europeans to East Asians reflects lower statistical power in the latter or differences in local LD structure across ancestries. In addition, statistical power can be limited in colocalization analysis, which can reduce the probability of shared causal variants across traits examined being detected (i.e., leading to “false negative” findings). We therefore cannot rule out the possibility that some colocalization findings suggesting low posterior probabilities of shared causal variants across traits (e.g., colocalization analyses for genetically proxied NCC inhibition and ER− breast cancer risk) reflected the limited power of this approach. Third, while we were able to perform sensitivity analyses for ADRB1 inhibition by restricting instruments to SNPs that were also eQTLs for ADRB1, we were unable to find evidence that the SNP used to instrument NCC (rs35797045) was also an eQTL for the gene encoding this target. Fourth, while these analyses did not account for previously reported associations of genetically proxied elevated SBP with antihypertensive medication use within the colorectal cancer datasets analyzed, such correction would be expected to strengthen, rather than attenuate, findings presented in this study [80]. Likewise, in “instrument validation” analyses for ADRB1, our inability to recapitulate known effects of ADRB1 inhibition (via beta blockers) on stroke risk could reflect the aforementioned inability to account for blood pressure medication users in the stroke datasets analyzed. It is also possible that these findings reflect the presence of horizontal pleiotropy in the instrument biasing the effect estimate toward the null and/or a nontarget-mediated effect of these medications on stroke risk. Fifth, though mixed-ancestry GWAS were used to construct instruments for serum ACE concentrations, effect allele frequencies for variants used in this instrument were similar across European and Latin American ancestry participants, suggesting that mendelian randomization findings were unlikely to be influenced by confounding through residual population stratification. Sixth, effect estimates presented make the additional assumptions of linearity and the absence of gene–environment and gene–gene interactions. Seventh, our genetically proxied ADRB1 findings are of greater relevance to second generation β blockers (e.g., atenolol and metoprolol), which selectively inhibit ADRB1 as compared to first generation β blockers (e.g., propranolol and nadolol), which equally inhibit ADRB1 and ADRB2 [81]. Future mendelian randomization analyses examining the potential effects of long-term first generation β blocker use incorporating both *ADRB1* and *ADRB2* variants is warranted. Eighth, we cannot rule out findings presented being influenced by canalization (i.e., compensatory processes being generated during development that counter the phenotypic impact of genetic variants being used as instruments). Finally, while various sensitivity analyses were performed to examine exchangeability and exclusion restriction violations, these assumptions are unverifiable.

Colorectal cancer is the third most common cause of cancer globally [82]. Given the prevalence of ACE inhibitor use in high- and middle-income countries and growing use in low-income countries, and the often long-term nature of antihypertensive therapy, these findings, if replicated in subsequent clinical trials, may have important implications for choice of antihypertensive therapy [4]. Importantly, given that hypertension is more prevalent among those who are overweight or obese (risk factors for colorectal cancer), these findings suggest that long-term use of this medication could increase colorectal cancer risk among populations who are already at elevated risk of this disease. There are different types and classes of ACE inhibitors, which vary in their pharmacodynamic and pharmacokinetic properties [83]. These differing pharmacological properties (e.g., differential absorption rate, affinity for tissue-bound ACE, and plasma half-life) can influence therapeutic benefit (or experience of adverse effects) of ACE inhibitors [84,85]. Future evaluation of the potential effects of long-term ACE inhibitor use on cancer risk should therefore include assessment of whether findings are specific to individual agents or classes of ACE inhibitors. As data on circulating levels of ADRB1 and NCC become available in future GWAS, there would be merit in evaluating whether findings presented in this analysis can be replicated when using alternate instruments developed from protein quantitative loci for these targets. Further work is warranted to unravel molecular mechanisms underpinning the association of ACE with colorectal cancer risk. In addition, extension of the analyses presented in this study to a survival framework could inform on whether concurrent use of ACE inhibitors may have an adverse effect on prognosis among colorectal cancer patients. Finally, findings from this analysis should be “triangulated” by employing other epidemiological designs with orthogonal (i.e., nonoverlapping) sources of bias to each other to further evaluate the association of ACE inhibition and colorectal cancer risk [86].

## Conclusions

Our mendelian randomization analyses suggest that genetically proxied long-term inhibition of ACE is associated with increased risk of colorectal cancer. Evaluation of ACE inhibitor use in randomized controlled trials with sufficient follow-up data can inform on the long-term safety of these medications. Our findings provide human genetic support to results from short-term randomized trials suggesting that long-term use of β blockers and thiazide diuretics may not influence risk of common cancers.

## Supporting information

S1 STROBE Statement STROBE-MR checklist of recommended items to address in reports of mendelian randomization studies. (DOCX)

S1 Table Characteristics of SBP lowering genetic variants in *ADRB1* in sensitivity analyses using a GWAS unadjusted for BMI or antihypertensive medication use. Footnote: No SNPs at genome-wide significance (*P* < 5 × 10^−8^) were available to instrument NCC. Effect (SE) represents change in SBP per additional copy of the effect allele. ADRB1, β-1 adrenergic receptor; BMI, body mass index; NCC, sodium-chloride symporter; SBP, systolic blood pressure; SNP, single-nucleotide polymorphism.
(DOCX)

S2 Table Comparison of effect allele frequency for variants included as ACE instruments across European and Latin American participants in serum ACE concentrations GWAS and colorectal cancer risk in participants of European ancestry. Footnote: ACE, angiotensin-converting enzyme; EUR, European participants; GWAS, genome-wide association study; LA, Latin American participants; SNP, single-nucleotide polymorphism.
(DOCX)

S3 Table Instrument strength estimates for drug target instruments. Footnote: Range represents r^2^ and F-stats across instruments applying a linkage disequilibrium threshold of <0.01 to <0.10.
(DOCX)

S4 Table Posterior probabilities under differing hypotheses relating the associations between serum ACE concentrations and colorectal cancer risk. Footnote: H_0_ = neither serum ACE concentrations nor colorectal cancer risk has a genetic association in the region, H_1_ = only serum ACE concentrations has a genetic association in the region, H_2_ = only colorectal cancer risk has a genetic association in the region, H_3_ = both serum ACE concentrations and colorectal cancer risk are associated but have different causal variants, H_4_ = both serum ACE concentrations and colorectal cancer risk are associated and share a single causal variant. ACE, angiotensin-converting enzyme.
(DOCX)

S5 Table Association between genetically proxied ACE inhibition and previously reported risk factors for colorectal cancer. Footnote: Effect represents the unit change in colorectal cancer risk factor per genetically proxied inhibition of ACE equivalent to a 1-mm Hg decrease in SBP. For analyses of genetically proxied ACE inhibition and low-density lipoprotein cholesterol, 2 SNPs (rs12452187 and rs11655956) were not available, and 1 SNP (rs118138685) was removed because of palindromic alleles with ambiguous effect allele frequencies not permitting strands to be matched. For analyses of iron, 1 SNP (rs11655956) was not included because of palindromic alleles with ambiguous effect allele frequencies. For analyses of insulin-like growth factor 1, 2 SNPs (rs11655956 and rs118138685) were not included because of palindromic alleles with ambiguous effect allele frequencies. For analyses of alcohol intake, 2 SNPs (rs12452187 and rs12150648) were not available in the outcome dataset. ACE, angiotensin-converting enzyme; SBP, systolic blood pressure; SNP, single-nucleotide polymorphism.
(DOCX)

S6 Table Association between genetically proxied ACE inhibition and colorectal cancer risk in iterative leave-one-out analysis. Footnote: OR represents the exponential change in odds of cancer per genetically proxied inhibition of ACE equivalent to a 1 mmHg decrease in SBP. ACE, angiotensin-converting enzyme; OR, odds ratio; SBP, systolic blood pressure; SNP, single-nucleotide polymorphism.
(DOCX)

S7 Table Posterior probabilities under differing hypotheses relating the associations between SBP (in *ADRB1*) and small cell lung carcinoma risk. Footnote: Marginal = SNP associations that are unconditioned (on either the sentinel SNP or additional conditionally independent genome-wide significant SNP for each respective trait), * = Sentinel SNP, ^†^ = Conditionally independent and significant (*P* < 5 × 10^−8^) SNP, H_0_ = neither SBP (in *ADRB1*) nor small cell lung carcinoma risk has a genetic association in the region, H_1_ = only SBP (in *ADRB1*) has a genetic association in the region, H_2_ = only small cell lung carcinoma risk has a genetic association in the region, H_3_ = both SBP (in *ADRB1*) and small cell lung carcinoma risk are associated but have different causal variants, H_4_ = both SBP (in *ADRB1*) and small cell lung carcinoma risk are associated and share a single causal variant. ADRB1, β-1 adrenergic receptor; SBP, systolic blood pressure; SNP, single-nucleotide polymorphism.
(DOCX)

S8 Table Association between genetically proxied ADRB1 inhibition and risk of overall and subtype-specific breast, colorectal, prostate, and lung cancer risk using instrument constructed from a GWAS unadjusted for BMI. Footnote: OR represents the exponential change in odds of cancer per genetically proxied inhibition of ADRB1 equivalent to a 1-mm Hg decrease in SBP. ADRB1, β-1 adrenergic receptor; BMI, body mass index; GWAS, genome-wide association study; OR, odds ratio; SBP, systolic blood pressure.
(DOCX)

S9 Table Look-up of eQTL status of SNPs used to instrument ADRB1 and NCC inhibition in GTEx V8, eQTLGen, and BarcUVa-Seq. Footnote: ADRB1, β-1 adrenergic receptor; EA, effect allele; eQTL, expression quantitative trait locus; NCC, sodium-chloride symporter; NEA, noneffect allele; SE, standard error; NES, normalized effect size (obtained from GTex V8); SNP, single-nucleotide polymorphism; TMMs, trimmed mean of M-values (obtained from Barc-UVa-Seq); Z, Z-statistic (obtained from blood EQTL gen).
(DOCX)

S10 Table Association between genetically proxied ADRB1 inhibition and risk of coronary artery disease, stroke, overall and subtype-specific breast, colorectal, prostate, and lung cancer risk using instruments that are also eQTLs for *ADRB1* expression. Footnote: Scaled to represent ADRB1 inhibition equivalent of a 1-mm Hg SBP reduction. * Neither rs1801253 (nor a high LD proxy) was available in PRACTICAL for prostate cancer risk. ADRB1, β-1 adrenergic receptor; eQTLs, expression quantitative trait loci; SBP, systolic blood pressure.
(DOCX)

S11 Table Posterior probabilities under differing hypotheses relating the associations between SBP (in *SLC12A3*) and ER− breast cancer risk. Footnote: Marginal = SNP associations that are unconditioned (on either the sentinel SNP or additional conditionally independent genome-wide significant SNP for each respective trait), * = Sentinel SNP, ^†^ = Conditionally independent and significant (*P* < 5 × 10^−8^) SNP, H_0_ = neither SBP (in *SLC12A3*) nor ER− breast cancer risk has a genetic association in the region, H_1_ = only SBP (in *SLC12A3*) has a genetic association in the region, H_2_ = only ER− breast cancer risk has a genetic association in the region, H_3_ = both SBP (in *SLC12A3*) and ER− breast cancer risk are associated but have different causal variants, H_4_ = both SBP (in *SLC12A3*) and ER− breast cancer risk are associated and share a single causal variant. ER, estrogen receptor; SBP, systolic blood pressure; SNP, single-nucleotide polymorphism.
(DOCX)

S12 Table Posterior probabilities under differing hypotheses relating the associations between colon ACE gene expression and colorectal cancer risk. Footnote: Marginal = SNP associations that are unconditioned (on either the sentinel SNP or additional conditionally independent genome-wide significant SNP for each respective trait), * = Sentinel SNP, ^†^ = Conditionally independent and significant (*P* < 5 × 10^−8^) SNP, H_0_ = neither colon ACE concentrations nor colorectal cancer risk has a genetic association in the region, H_1_ = only colon ACE concentrations has a genetic association in the region, H_2_ = only colorectal cancer risk has a genetic association in the region, H_3_ = both colon ACE concentrations and colorectal cancer risk are associated but have different causal variants, H_4_ = both colon ACE concentrations and colorectal cancer risk are associated and share a single causal variant. ACE, angiotensin-converting enzyme; SNP, single-nucleotide polymorphism.
(DOCX)

S13 Table Transcriptome-wide GRS analysis. Caption: gene_id = ENSEMBL gene id, beta = effect estimate, se = standard error, pval (not adjusted for multiple testing), R2 = proportion of variance explained by serum ACE wGRS, z-score = test-statistic for association between serum ACE wGRS and gene expression, p.adjust = p-value adjusted for multiple testing using a Bonferroni correction, chr = chromosome of gene, start = location of start of gene, end = location of end of gene, strand = coding strand, gene_type = type of gene examined. ACE, angiotensin-converting enzyme; GRS, genetic risk score; wGRS, weighted genetic risk score.
(XLSX)

S14 Table Gene set enrichment analysis of genes whose expression was associated with genetically proxied serum ACE inhibition (at *P* value < 5.00 × 10^−3^). Caption: Category = one of 9 “major collections” included in the MSigDB, GeneSet = name of gene set as provided by MSigDB, N_genes = number of genes in gene set, N_overlap = number of overlapping genes from wGRS analysis in gene set, p = p-value (unadjusted for multiple testing), adjP = p-value (adjusted for multiple testing), genes = genes from wGRS analysis that overlap with gene set, link = link to further information on gene set. ACE, angiotensin-converting enzyme; MSigDB, Molecular Signatures Database; wGRS, weighted genetic risk score.
(XLSX)

S15 Table Genes from the black module identified in the coexpression network analysis. Caption: N/A.
(XLSX)

S16 Table Gene set enrichment analysis of genes from black module. Caption: Category = one of 9 “major collections” included in the MSigDB, GeneSet = name of gene set as provided by MSigDB, N_genes = number of genes in gene set, N_overlap = number of genes located in the black module of the coexpression network in gene set, p = p-value (unadjusted for multiple testing), adjP = p-value (adjusted for multiple testing), genes = genes from black module of the coexpression network that overlap with gene set, link = link to further information on gene set. MSigDB, Molecular Signatures Database.
(XLSX)

S1 Text Cancer consortia-specific funding and acknowledgments. (DOCX)

S1 Methods Colocalization analysis. (DOCX)

S2 Methods Sensitivity analyses using eQTL data. (DOCX)

S3 Methods BarcUVa-Seq study. (DOCX)

S4 Methods Coexpression network and gene set enrichment analyses. (DOCX)

## Abbreviations

ACE: angiotensin-converting enzyme
ADRB1: β-1 adrenergic receptor
BCAC: Breast Cancer Association Consortium
BMI: body mass index
CCFR: Colon Cancer Family Registry
CORECT: ColoRectal Transdisciplinary Study
eQTLs: expression quantitative trait loci
ER: estrogen receptor
GECCO: Genetics and Epidemiology of Colorectal Cancer Consortium
GERA: Genetic Epidemiology Research on Adult Health and Aging
GWAS: genome-wide association study
ICBP: International Consortium of Blood Pressure
ILCCO: International Lung Cancer Consortium
INTEGRAL: Integrative Analysis of Lung Cancer Risk and Etiology
NCC: sodium-chloride symporter
OR: odds ratio
ORIGIN: Outcome Reduction with Initial Glargine Intervention
PEER: probabilistic estimation of expression residuals
PRACTICAL: Prostate Cancer Association Group to Investigate Cancer Associated Alterations in the Genome consortium
RAS: renin–angiotensin system
SBP: systolic blood pressure
SNP: single-nucleotide polymorphism
TMM: trimmed mean of M-values
wGRS: weighted genetic risk score
