# Supplementary material for: Genetically proxied therapeutic inhibition of antihypertensive drug targets and risk of common cancers: A mendelian randomization analysis
Source: PLoS Med. 2022 Feb 3;19(2):e1003897. doi: 10.1371/journal.pmed.1003897 (PMC8812899; doi:10.1371/journal.pmed.1003897)
Supplement: S1 Table — Footnote: No SNPs at genome-wide significance (P < 5 × 10^−8^) were available to instrument NCC. Effect (SE) represents change in SBP per additional copy of the effect allele. ADRB1, β-1 adrenergic receptor; BMI, body mass index; NCC, sodium-chloride symporter; SBP, systolic blood pressure; SNP, single-nucleotide polymorphism. (DOCX) [file pmed.1003897.s002.docx]

S1 Table. Characteristics of systolic blood pressure lowering genetic variants in *ADRB1* in sensitivity analyses using a GWAS unadjusted for body mass index or antihypertensive medication use

| **SNP** | **Effect Allele/Non-Effect Allele** | **Effect Allele Frequency** | **Effect (SE)** | ***P*-value** |
| --- | --- | --- | --- | --- |
| rs2782980 | T/C | 0.28 | -0.35 (0.05) | 1.27 x 10^-13^ |
| rs180940 | A/G | 0.33 | -0.30 (0.04) | 4.39 x 10^-11^ |

SNP = Single-Nucleotide Polymorphism. No SNPs at genome-wide significance (*P* < 5 x 10^-8^) were available to instrument NCC. Effect (SE) represents change in systolic blood pressure per additional copy of the effect allele.
