# Supplementary material for: Genetically proxied therapeutic inhibition of antihypertensive drug targets and risk of common cancers: A mendelian randomization analysis
Source: PLoS Med. 2022 Feb 3;19(2):e1003897. doi: 10.1371/journal.pmed.1003897 (PMC8812899; doi:10.1371/journal.pmed.1003897)
Supplement: S2 Table — Footnote: ACE, angiotensin-converting enzyme; EUR, European participants; GWAS, genome-wide association study; LA, Latin American participants; SNP, single-nucleotide polymorphism. (DOCX) [file pmed.1003897.s003.docx]

S2 Table. Comparison of effect allele frequency for variants included as ACE instruments across European and Latin American participants in serum ACE concentrations genome-wide association study and colorectal cancer risk in participants of European ancestry

| **SNP** | **Effect Allele** | **Serum ACE (EUR)** | **Serum ACE**  **(LA)** | **Colorectal cancer (EUR)** |
| --- | --- | --- | --- | --- |
| rs4343 | A | 0.45 | 0.46 | 0.47 |
| rs12452187 | A | 0.60 | 0.61 | 0.62 |
| rs79480822 | C | 0.93 | 0.97 | 0.94 |
| rs3730025 | G | 0.01 | 0.01 | 0.01 |
| rs4365 | G | 0.97 | 0.98 | 0.96 |
| rs80311894 | T | 0.97 | 0.97 | 0.95 |
| rs4968780 | C | 0.05 | 0.06 | 0.04 |
| rs11655956 | C | 0.08 | 0.08 | 0.09 |
| rs118121655 | G | 0.96 | 0.97 | 0.97 |
| rs28656895 | T | 0.23 | 0.24 | 0.23 |
| Rs4968771 | G | 0.08 | 0.12 | 0.09 |
| Rs12150648 | G | 0.96 | 0.97 | 0.97 |
| Rs118138685 | C | 0.04 | 0.02 | 0.05 |
| Rs13342595 | C | 0.23 | 0.24 | 0.23 |

SNP = Single-Nucleotide Polymorphism, ACE = Angiotensin-converting enzyme, EUR = European participants, LA = Latin American participants.
