# Supplementary material for: Genetically proxied therapeutic inhibition of antihypertensive drug targets and risk of common cancers: A mendelian randomization analysis
Source: PLoS Med. 2022 Feb 3;19(2):e1003897. doi: 10.1371/journal.pmed.1003897 (PMC8812899; doi:10.1371/journal.pmed.1003897)
Supplement: S3 Table — Footnote: Range represents r2 and F-stats across instruments applying a linkage disequilibrium threshold of <0.01 to <0.10. (DOCX) [file pmed.1003897.s004.docx]

Table S3. Instrument strength estimates for drug target instruments

| **Target** | **N of SNPs** | **R^2^** | **F-stats** |
| --- | --- | --- | --- |
| ACE | 3-14 | 0.34-0.39 | 2,156.5-2,594.9 |
| ADRB1 | 2-8 | 0.00031-0.00067 | 269.1-572.2 |
| NCC | 1 | 0.0021 | 1659.9 |

Range represents r^2^ and F-stats across instruments applying a linkage disequilibrium threshold of < 0.01 to < 0.10.
