# Supplementary material for: Genetically proxied therapeutic inhibition of antihypertensive drug targets and risk of common cancers: A mendelian randomization analysis
Source: PLoS Med. 2022 Feb 3;19(2):e1003897. doi: 10.1371/journal.pmed.1003897 (PMC8812899; doi:10.1371/journal.pmed.1003897)
Supplement: S4 Table — Footnote: H0 = neither serum ACE concentrations nor colorectal cancer risk has a genetic association in the region, H1 = only serum ACE concentrations has a genetic association in the region, H2 = only colorectal cancer risk has a genetic association in the region, H3 = both serum ACE concentrations and colorectal cancer risk are associated but have different causal variants, H4 = both serum ACE concentrations and colorectal cancer risk are associated and share a single causal variant. ACE, angiotensin-converting enzyme. (DOCX) [file pmed.1003897.s005.docx]

S4 Table. Posterior probabilities under differing hypotheses relating the associations between serum ACE concentrations and colorectal cancer risk

| **Configuration** | **H_0_** | **H_1_** | **H_2_** | **H_3_** | **H_4_** |
| --- | --- | --- | --- | --- | --- |
|  | 1.27 x 10^-230^ | 6.16 x 10^-2^ | 5.12 x 10^-231^ | 2.40 x 10^-2^ | 0.91 |

H_0_ = neither serum ACE concentrations nor colorectal cancer risk has a genetic association in the region, H_1_ = only serum ACE concentrations has a genetic association in the region, H_2_ = only colorectal cancer risk has a genetic association in the region, H_3_ = both serum ACE concentrations and colorectal cancer risk are associated but have different causal variants, H_4_= both serum ACE concentrations and colorectal cancer risk are associated and share a single causal variant
