# Supplementary material for: Genetically proxied therapeutic inhibition of antihypertensive drug targets and risk of common cancers: A mendelian randomization analysis
Source: PLoS Med. 2022 Feb 3;19(2):e1003897. doi: 10.1371/journal.pmed.1003897 (PMC8812899; doi:10.1371/journal.pmed.1003897)
Supplement: S5 Table — Footnote: Effect represents the unit change in colorectal cancer risk factor per genetically proxied inhibition of ACE equivalent to a 1-mm Hg decrease in SBP. For analyses of genetically proxied ACE inhibition and low-density lipoprotein cholesterol, 2 SNPs (rs12452187 and rs11655956) were not available, and 1 SNP (rs118138685) was removed because of palindromic alleles with ambiguous effect allele frequencies not permitting strands to be matched. For analyses of iron, 1 SNP (rs11655956) was not included because of palindromic alleles with ambiguous effect allele frequencies. For analyses of insulin-like growth factor 1, 2 SNPs (rs11655956 and rs118138685) were not included because of palindromic alleles with ambiguous effect allele frequencies. For analyses of alcohol intake, 2 SNPs (rs12452187 and rs12150648) were not available in the outcome dataset. ACE, angiotensin-converting enzyme; SBP, systolic blood pressure; SNP, single-nucleotide polymorphism. (DOCX) [file pmed.1003897.s006.docx]

S5 Table. Association between genetically-proxied ACE inhibition and previously reported risk factors for colorectal cancer

| **Previously reported risk factor** | **N** | **Effect estimate**  **(95% CI)** | ***P*-value** |
| --- | --- | --- | --- |
| Body mass index (SD) | 461,460 | -0.018 (-0.042, 0.007) | 0.11 |
| Low density-lipoprotein cholesterol (SD, mg/dL) | 9,961 | 0.050 (-0.024, 0.124) | 0.18 |
| Serum total cholesterol (SD) | 21,491 | -0.040 (-0.109, 0.029) | 0.23 |
| Serum iron (SD, µmol/L) | 23,896 | -0.020 (-0.094, 0.054) | 0.61 |
| Insulin-like growth factor-1 (Nmol/L) | 9,732 | 3.297 (-1.131, 7.726) | 0.15 |
| Alcohol intake (SD, log-alcoholic drinks/week) | 335,394 | 0.005 (-0.008, 0.018) | 0.45 |
| Standing height (SD) | 461,950 | 0.022 (-0.006, 0.049) | 0.12 |
| Physical activity (SD) | 91,105 | -0.018 (-0.047, 0.012) | 0.25 |

Effect represents the unit change in colorectal cancer risk factor per genetically proxied inhibition of ACE equivalent to a 1 mmHg decrease in systolic blood pressure.

For analyses of genetically-proxied ACE inhibition and low-density lipoprotein cholesterol, two SNPs (rs12452187, rs11655956) were not available and 1 SNP (rs118138685) was removed because of palindromic alleles with ambiguous effect allele frequencies not permitting strands to be matched. For analyses of iron, 1 SNP (rs11655956) was not included because of palindromic alleles with ambiguous effect allele frequencies. For analyses of insulin-like growth factor 1, 2 SNPs (rs11655956, rs118138685) were not included because of palindromic alleles with ambiguous effect allele frequencies. For analyses of alcohol intake, 2 SNPs (rs12452187, rs12150648) were not available in the outcome dataset.
