# Supplementary material for: Genetically proxied therapeutic inhibition of antihypertensive drug targets and risk of common cancers: A mendelian randomization analysis
Source: PLoS Med. 2022 Feb 3;19(2):e1003897. doi: 10.1371/journal.pmed.1003897 (PMC8812899; doi:10.1371/journal.pmed.1003897)
Supplement: S6 Table — Footnote: OR represents the exponential change in odds of cancer per genetically proxied inhibition of ACE equivalent to a 1 mmHg decrease in SBP. ACE, angiotensin-converting enzyme; OR, odds ratio; SBP, systolic blood pressure; SNP, single-nucleotide polymorphism. (DOCX) [file pmed.1003897.s007.docx]

S6 Table. Association between genetically-proxied ACE inhibition and colorectal cancer risk in iterative leave-one-out analysis

| **SNP removed** | **OR (95% CI)** | ***P*-value** |
| --- | --- | --- |
| rs11650201 | 1.13 (1.05-1.22) | 8.6 x 10^-4^ |
| rs11655956 | 1.14 (1.06-1.22) | 2.7 x 10^-4^ |
| rs118121655 | 1.14 (1.06-1.23) | 5.3 x 10^-4^ |
| rs12452187 | 1.13 (1.05-1.22) | 8.6 x 10^-4^ |
| rs12709437 | 1.13 (1.05-1.22) | 1.1 x 10^-3^ |
| rs141118688 | 1.13 (1.05-1.22) | 8.6 x 10^-4^ |
| rs28656895 | 1.14 (1.07-1.22) | 6.3 x 10^-5^ |
| rs3730025 | 1.14 (1.06-1.22) | 2.7 x 10^-4^ |
| rs4343 | 1.12 (1.02-1.23) | 1.8 x 10^-2^ |
| rs4365 | 1.13 (1.05-1.22) | 1.1 x 10^-3^ |
| rs4968780 | 1.13 (1.05-1.21) | 6.1 x 10^-4^ |
| rs79480822 | 1.14 (1.06-1.22) | 6.7 x 10^-4^ |
| rs80311894 | 1.14 (1.06-1.22) | 6.7 x 10^-4^ |

SNP = Single-Nucleotide Polymorphism. OR represents the exponential change in odds of cancer per genetically proxied inhibition of ACE equivalent to a 1 mmHg decrease in systolic blood pressure.
