# Supplementary material for: Genetically proxied therapeutic inhibition of antihypertensive drug targets and risk of common cancers: A mendelian randomization analysis
Source: PLoS Med. 2022 Feb 3;19(2):e1003897. doi: 10.1371/journal.pmed.1003897 (PMC8812899; doi:10.1371/journal.pmed.1003897)
Supplement: S7 Table — Footnote: Marginal = SNP associations that are unconditioned (on either the sentinel SNP or additional conditionally independent genome-wide significant SNP for each respective trait), * = Sentinel SNP, † = Conditionally independent and significant (P < 5 × 10^−8^) SNP, H0 = neither SBP (in ADRB1) nor small cell lung carcinoma risk has a genetic association in the region, H1 = only SBP (in ADRB1) has a genetic association in the region, H2 = only small cell lung carcinoma risk has a genetic association in the region, H3 = both SBP (in ADRB1) and small cell lung carcinoma risk are associated but have different causal variants, H4 = both SBP (in ADRB1) and small cell lung carcinoma risk are associated and share a single causal variant. ADRB1, β-1 adrenergic receptor; SBP, systolic blood pressure; SNP, single-nucleotide polymorphism. (DOCX) [file pmed.1003897.s008.docx]

S7 Table. Posterior probabilities under differing hypotheses relating the associations between systolic blood pressure (in *ADRB1*) and small cell lung carcinoma risk

| **SBP SNP** | **Small cell lung carcinoma SNP** | **H_0_** | **H_1_** | **H_2_** | **H_3_** | **H_4_** |
| --- | --- | --- | --- | --- | --- | --- |
| Marginal | Marginal | 5.11 x 10^-36^ | 0.73 | 1.76 x 10^-36^ | 0.25 | 1.53 x 10^-2^ |
| rs1801253* | Marginal | 1.57 x 10^-39^ | 0.73 | 5.37 x 10^-40^ | 0.25 | 1.37 x 10^-2^ |
| rs462536^†^ | Marginal | 9.18 x 10^-36^ | 0.73 | 3.15 x 10^-36^ | 0.25 | 1.71 x 10^-2^ |
| rs4918889^†^ | Marginal | 9.18 x 10^-36^ | 0.73 | 3.15 x 10^-36^ | 0.25 | 1.71 x 10^-2^ |
| rs6585252^†^ | Marginal | 9.18 x 10^-36^ | 0.73 | 3.15 x 10^-36^ | 0.25 | 1.71 x 10^-2^ |

Marginal = SNP associations that are unconditioned (on either the sentinel SNP or additional conditionally independent genome-wide significant SNP for each respective trait), * = Sentinel SNP, ^†^ = Conditionally independent and significant (P<5x10^-8^) SNP, H_0_ = neither systolic blood pressure (in *ADRB1*) nor small cell lung carcinoma risk has a genetic association in the region, H_1_ = only systolic blood pressure (in *ADRB1*) has a genetic association in the region, H_2_ = only small cell lung carcinoma risk has a genetic association in the region, H_3_ = both systolic blood pressure (in *ADRB1*) and small cell lung carcinoma risk are associated but have different causal variants, H_4_= both systolic blood pressure (in *ADRB1*) and small cell lung carcinoma risk are associated and share a single causal variant
