# Supplementary material for: Genetically proxied therapeutic inhibition of antihypertensive drug targets and risk of common cancers: A mendelian randomization analysis
Source: PLoS Med. 2022 Feb 3;19(2):e1003897. doi: 10.1371/journal.pmed.1003897 (PMC8812899; doi:10.1371/journal.pmed.1003897)
Supplement: S8 Table — Footnote: OR represents the exponential change in odds of cancer per genetically proxied inhibition of ADRB1 equivalent to a 1-mm Hg decrease in SBP. ADRB1, β-1 adrenergic receptor; BMI, body mass index; GWAS, genome-wide association study; OR, odds ratio; SBP, systolic blood pressure. (DOCX) [file pmed.1003897.s009.docx]

S8 Table. Association between genetically-proxied ADRB1 inhibition and risk of overall and subtype-specific breast, colorectal, prostate, and lung cancer risk using instrument constructed from a GWAS unadjusted for BMI

| **Outcome** | **N (cases, controls)** | **OR (95% CI)** | ***P*-value** |
| --- | --- | --- | --- |
| Breast cancer | 122,977; 105,974 | 1.04 (1.01-1.07) | 0.02 |
| ER+ Breast cancer | 69,501; 105,974 | 1.04 (1.00-1.08) | 0.06 |
| ER- Breast cancer | 21,468; 105,974 | 1.00 (0.94-1.06) | 0.89 |
|  |  |  |  |
| Colorectal cancer | 58,221; 67,694 | 0.99 (0.95-1.04) | 0.71 |
| Colon cancer | 32,002; 64,159 | 1.00 (0.95-1.05) | 0.87 |
| Rectal cancer | 16,212; 64,159 | 1.02 (0.95-1.08) | 0.67 |
|  |  |  |  |
| Lung cancer | 29,863; 55,586 | 1.01 (0.94-1.08) | 0.87 |
| Lung adenocarcinoma | 11,245; 54,619 | 0.97 (0.89-1.05) | 0.41 |
| Small cell lung carcinoma | 2,791; 20,580 | 0.88 (0.77-1.02) | 0.08 |
| Squamous cell lung cancer | 7,704; 54,763 | 0.98 (0.89-1.07) | 0.63 |
|  |  |  |  |
| Prostate cancer | 79,148; 61,106 | 0.99 (0.95-1.03) | 0.70 |
| Advanced prostate cancer | 15,167; 58,308 | 0.97 (0.91-1.04) | 0.43 |

OR represents the exponential change in odds of cancer per genetically proxied inhibition of ADRB1 equivalent to a 1 mmHg decrease in systolic blood pressure.
