# Supplementary material for: Genetically proxied therapeutic inhibition of antihypertensive drug targets and risk of common cancers: A mendelian randomization analysis
Source: PLoS Med. 2022 Feb 3;19(2):e1003897. doi: 10.1371/journal.pmed.1003897 (PMC8812899; doi:10.1371/journal.pmed.1003897)
Supplement: S9 Table — Footnote: ADRB1, β-1 adrenergic receptor; EA, effect allele; eQTL, expression quantitative trait locus; NCC, sodium-chloride symporter; NEA, noneffect allele; SE, standard error; NES, normalized effect size (obtained from GTex V8); SNP, single-nucleotide polymorphism; TMMs, trimmed mean of M-values (obtained from Barc-UVa-Seq); Z, Z-statistic (obtained from blood EQTL gen). (DOCX) [file pmed.1003897.s010.docx]

S9 Table. Look-up of eQTL status of SNPs used to instrument ADRB1 and NCC inhibition in GTEx V8, eQTLGen, and BarcUVa-Seq.

| **Target** | **EA/**  **NEA** | **Effect (SE)**  **(SBP)** | ***P*-value**  **(SBP)** | **Tissue**  **(gene expression)** | **Beta**  **(gene expression)** | ***P*-value**  **(gene expression)** |
| --- | --- | --- | --- | --- | --- | --- |
| *ADRB1* |  |  |  |  |  |  |
| rs1801253 | G/C | -0.41 (0.03) | 8.07x10^-43^ | Testis | -0.46 (NES) | 3.5 x 10^-13^ |
| rs11196549 | G/A | -0.62 (0.07) | 2.53x10^-19^ | Whole blood | 4.37  (Z) | 1.2 x 10^-5^ |
| rs4918889 | G/C | -0.30 (0.04) | 7.53x10^-18^ | Whole blood | 18.43 (NES) | 7.0 x 10^-76^ |
|  |  |  |  | Colon | -0.31  (TMM) | 4.8 x 10^-5^ |
|  |  |  |  | Muscle (Skeletal) | -0.31 (NES) | 2.5 x 10^-6^ |
| rs460718 | A/G | -0.24 (0.03) | 2.21x10^-17^ | - | - | - |
| rs11196597 | G/A | -0.27 (0.04) | 3.07x10^-12^ | - | - | - |
| rs143854972 | G/A | -0.39 (0.06) | 4.35x10^-11^ | Whole blood | -10.73 (Z) | 7.7 x 10^-27^ |
| rs17875473 | C/T | -0.28 (0.05) | 9.04x10^-9^ | Whole blood | 6.3  (Z) | 2.7 x 10^-10^ |
| rs10787510 | A/G | -0.15 (0.03) | 2.01x10^-8^ | Whole blood | -5.48 (Z) | 4.3 x 10^-8^ |
|  |  |  |  | Pancreas | -0.31 (NES) | 2.0 x 10^-5^ |
|  |  |  |  |  |  |  |
| *NCC* |  |  |  |  |  |  |
| rs35797045 | A/C | -0.35 (0.06) | 4.85x10^-8^ | - | - | - |

EA = Effect Allele, NEA = Non-Effect Allele, SE = Standard Error, NES = Normalized Effect Size (obtained from GTex V8), Z = Z-statistic (obtained from blood EQTL gen), TMMs = Trimmed Mean of M-values (obtained from Barc-UVa-Seq).
