# Supplementary material for: Genetically proxied therapeutic inhibition of antihypertensive drug targets and risk of common cancers: A mendelian randomization analysis
Source: PLoS Med. 2022 Feb 3;19(2):e1003897. doi: 10.1371/journal.pmed.1003897 (PMC8812899; doi:10.1371/journal.pmed.1003897)
Supplement: S10 Table — Footnote: Scaled to represent ADRB1 inhibition equivalent of a 1-mm Hg SBP reduction. * Neither rs1801253 (nor a high LD proxy) was available in PRACTICAL for prostate cancer risk. ADRB1, β-1 adrenergic receptor; eQTLs, expression quantitative trait loci; SBP, systolic blood pressure. (DOCX) [file pmed.1003897.s011.docx]

S10 Table. Association between genetically-proxied ADRB1 inhibition and risk of coronary artery disease, stroke, overall and subtype-specific breast, colorectal, prostate, and lung cancer risk using instruments that are also eQTLs for *ADRB1* expression

| **Outcome** | **OR (95% CI)** | ***P*-value** |
| --- | --- | --- |
| Coronary artery disease (8 SNP) | 0.95 (0.92-0.98) | 1.5 x 10^-3^ |
| Coronary artery disease (3 SNP) | 0.96 (0.91-1.00) | 0.06 |
| Stroke (8 SNP) | 1.03 (0.99-1.07) | 0.18 |
| Stroke (3 SNP) | 1.01 (0.96-1.06) | 0.68 |
|  |  |  |
| Breast cancer (8 SNP) | 1.01 (0.99-1.04) | 0.38 |
| Breast cancer (3 SNP) | 1.01 (0.98-1.04) | 0.56 |
| ER+ Breast cancer (8 SNP) | 1.01 (0.98-1.04) | 0.44 |
| ER+ Breast cancer (3 SNP) | 1.02 (0.99-1.06) | 0.24 |
| ER- Breast cancer (8 SNP) | 0.98 (0.94-1.02) | 0.38 |
| ER- Breast cancer (3 SNP) | 0.97 (0.91-1.03) | 0.29 |
|  |  |  |
| Colorectal cancer (8 SNP) | 0.98 (0.96-1.01) | 0.31 |
| Colorectal cancer (3 SNP) | 0.95 (0.91-1.00) | 0.04 |
| Colon cancer (8 SNP) | 0.99 (0.95-1.03) | 0.63 |
| Colon cancer (3 SNP) | 0.96 (0.91-1.02) | 0.18 |
| Rectal cancer (8 SNP) | 1.00 (0.95-1.04) | 0.84 |
| Rectal cancer (3 SNP) | 0.94 (0.88-1.01) | 0.09 |
|  |  |  |
| Lung cancer (8 SNP) | 1.01 (0.96-1.07) | 0.64 |
| Lung cancer (3 SNP) | 1.01 (0.94-1.09) | 0.73 |
| Lung adenocarcinoma (8 SNP) | 0.98 (0.91-1.04) | 0.48 |
| Lung adenocarcinoma (3 SNP) | 1.00 (0.92-1.09) | 0.94 |
| Small cell lung carcinoma (8 SNP) | 0.87 (0.79-0.96) | 0.008 |
| Small cell lung carcinoma (8 SNP) | 0.89 (0.77-1.03) | 0.13 |
| Squamous cell lung cancer (8 SNP) | 0.98 (0.91-1.06) | 0.67 |
| Squamous cell lung cancer (3 SNP) | 0.94 (0.85-1.03) | 0.16 |
|  |  |  |
| Prostate cancer (8 SNP) | 1.00 (0.96-1.03) | 0.73 |
| Prostate cancer (2 SNP)* | 1.05 (0.97-1.13) | 0.24 |
| Advanced prostate cancer (8 SNP) | 1.00 (0.94-1.06) | 0.97 |
| Advanced prostate cancer (3 SNP) | 1.02 (0.95-1.10) | 0.51 |

Scaled to represent ADRB1 inhibition equivalent of a 1 mmHg SBP reduction. * Neither rs1801253 (nor a high LD proxy) was available in PRACTICAL for prostate cancer risk.
