# Supplementary material for: Genetically proxied therapeutic inhibition of antihypertensive drug targets and risk of common cancers: A mendelian randomization analysis
Source: PLoS Med. 2022 Feb 3;19(2):e1003897. doi: 10.1371/journal.pmed.1003897 (PMC8812899; doi:10.1371/journal.pmed.1003897)
Supplement: S11 Table — Footnote: Marginal = SNP associations that are unconditioned (on either the sentinel SNP or additional conditionally independent genome-wide significant SNP for each respective trait), * = Sentinel SNP, † = Conditionally independent and significant (P < 5 × 10^−8^) SNP, H0 = neither SBP (in SLC12A3) nor ER− breast cancer risk has a genetic association in the region, H1 = only SBP (in SLC12A3) has a genetic association in the region, H2 = only ER− breast cancer risk has a genetic association in the region, H3 = both SBP (in SLC12A3) and ER− breast cancer risk are associated but have different causal variants, H4 = both SBP (in SLC12A3) and ER− breast cancer risk are associated and share a single causal variant. ER, estrogen receptor; SBP, systolic blood pressure; SNP, single-nucleotide polymorphism. (DOCX) [file pmed.1003897.s012.docx]

S11 Table. Posterior probabilities under differing hypotheses relating the associations between systolic blood pressure (in *SLC12A3*) and ER- breast cancer risk

| **SBP SNP** | **ER- breast cancer SNP** | **H_0_** | **H_1_** | **H_2_** | **H_3_** | **H_4_** |
| --- | --- | --- | --- | --- | --- | --- |
| Marginal | Marginal | 4.33x10^-2^ | 0.82 | 3.95 x 10^-3^ | 7.5 x 10^-2^ | 5.56 x 10^-2^ |
| rs35797045* | Marginal | 0.10 | 0.71 | 9.04 x 10^-3^ | 6.32 x 10^-2^ | 0.12 |

Marginal = SNP associations that are unconditioned (on either the sentinel SNP or additional conditionally independent genome-wide significant SNP for each respective trait), * = Sentinel SNP, ^†^ = Conditionally independent and significant (P<5x10^-8^) SNP, H_0_ = neither systolic blood pressure (in *SLC12A3*) nor ER- breast cancer risk has a genetic association in the region, H_1_ = only systolic blood pressure (in *SLC12A3*) has a genetic association in the region, H_2_ = only ER- breast cancer risk has a genetic association in the region, H_3_ = both systolic blood pressure (in *SLC12A3*) and ER- breast cancer risk are associated but have different causal variants, H_4_= both systolic blood pressure (in *SLC12A3*) and ER- breast cancer risk are associated and share a single causal variant
