# Supplementary material for: Genetically proxied therapeutic inhibition of antihypertensive drug targets and risk of common cancers: A mendelian randomization analysis
Source: PLoS Med. 2022 Feb 3;19(2):e1003897. doi: 10.1371/journal.pmed.1003897 (PMC8812899; doi:10.1371/journal.pmed.1003897)
Supplement: S12 Table — Footnote: Marginal = SNP associations that are unconditioned (on either the sentinel SNP or additional conditionally independent genome-wide significant SNP for each respective trait), * = Sentinel SNP, † = Conditionally independent and significant (P < 5 × 10^−8^) SNP, H0 = neither colon ACE concentrations nor colorectal cancer risk has a genetic association in the region, H1 = only colon ACE concentrations has a genetic association in the region, H2 = only colorectal cancer risk has a genetic association in the region, H3 = both colon ACE concentrations and colorectal cancer risk are associated but have different causal variants, H4 = both colon ACE concentrations and colorectal cancer risk are associated and share a single causal variant. ACE, angiotensin-converting enzyme; SNP, single-nucleotide polymorphism. (DOCX) [file pmed.1003897.s013.docx]

S12 Table. Posterior probabilities under differing hypotheses relating the associations between colon ACE gene expression and colorectal cancer risk

| **Colon ACE expression SNP** | **Colorectal cancer SNP** | **H_0_** | **H_1_** | **H_2_** | **H_3_** | **H_4_** |
| --- | --- | --- | --- | --- | --- | --- |
| Marginal | Marginal | 6.37x10^-268^ | 5.92x10^-268^ | 0.40 | 0.37 | 0.23 |
| Marginal | rs4291* | 0 | 0.47 | 0 | 0.24 | 0.29 |
| Marginal | rs112901380^†^ | 7.90x10^-246^ | 0.48 | 3.82x10^-246^ | 0.23 | 0.29 |
| Marginal | rs12452105^†^ | 7.90x10^-246^ | 0.48 | 3.83x10^-246^ | 0.23 | 0.29 |
| Marginal | rs1800764^†^ | 7.64x10^-246^ | 0.46 | 4.24x10^-246^ | 0.26 | 0.28 |
| Marginal | rs28710772^†^ | 7.90x10^-246^ | 0.48 | 3.83x10^-246^ | 0.23 | 0.29 |
| Marginal | rs4311^†^ | 7.44x10^-246^ | 0.45 | 4.57x10^-246^ | 0.28 | 0.27 |
| Marginal | rs4333^†^ | 7.54x10^-246^ | 0.46 | 4.41x10^-246^ | 0.27 | 0.28 |
| Marginal | rs4517853^†^ | 7.90x10^-246^ | 0.48 | 3.82x10^-246^ | 0.23 | 0.29 |

Marginal = SNP associations that are unconditioned (on either the sentinel SNP or additional conditionally independent genome-wide significant SNP for each respective trait), * = Sentinel SNP, ^†^ = Conditionally independent and significant (P<5x10^-8^) SNP, H_0_ = neither colon ACE concentrations nor colorectal cancer risk has a genetic association in the region, H_1_ = only colon ACE concentrations has a genetic association in the region, H_2_ = only colorectal cancer risk has a genetic association in the region, H_3_ = both colon ACE concentrations and colorectal cancer risk are associated but have different causal variants, H_4_= both colon ACE concentrations and colorectal cancer risk are associated and share a single causal variant
