# Supplementary material for: Genetically proxied therapeutic inhibition of antihypertensive drug targets and risk of common cancers: A mendelian randomization analysis
Source: PLoS Med. 2022 Feb 3;19(2):e1003897. doi: 10.1371/journal.pmed.1003897 (PMC8812899; doi:10.1371/journal.pmed.1003897)
Supplement: S1 Methods — (DOCX) [file pmed.1003897.s019.docx]

**S1 Methods. Colocalization analysis**

Colocalization tests the probability of shared causal variants between two (or more) traits. The presence of shared causal variants– as opposed to distinct causal variants that are in linkage disequilibrium with each other - is necessary in order to infer potential causality between these traits (though it is not sufficient as it does not account for horizontal pleiotropy and cannot inform on direction of association between traits). In order to examine whether there was evidence of colocalization for Mendelian randomization analyses showing nominal evidence of association (*P* < 0.05), we used the coloc package to quantify the probability of shared causal variants across SNP-drug target and SNP-cancer endpoint analyses. This package uses approximate Bayes factor (ABF) computation to generates posterior probabilities that associations between two traits represent each of the following configurations: (i) neither trait has a genetic association in the region (H_0_), (ii) only the first trait has a genetic association in the region (H_1_), (iii) only the second trait has a genetic association in the region (H_2_), (iv) both traits are associated but have different causal variants (H_3_) and (v) both traits are associated and share a single causal variant (H_4_). Colocalisation analysis was performed by generating ± 300 kb windows from the top SNP used to instrument each drug target. As a convention, a posterior probability of ≥ 0.80 was used to indicate support for a configuration tested.

Where there was insufficient support for two traits sharing a causal variant (posterior probability of H_4_ < 0.80) we then performed conditional analysis using GCTA-COJO and pairwise conditional analysis using the “coloc” R package as implemented in PWCoCo (https://github.com/jwr-git/pwcoco). Briefly, this method conditions associations for a particular trait on the sentinel SNP (i.e. SNP with smallest *P*-value that is < 5 x 10^-8^) to identify additional conditionally independent genome-wide significant (*P* < 5 x 10^-8^) SNPs associated with a trait of interest. Pairwise colocalisation analysis is then re-performed using association statistics for SNPs conditioned on either the sentinel SNP (across either trait) or additional conditionally independent SNPs (across either trait).
