# Supplementary material for: Genetically proxied therapeutic inhibition of antihypertensive drug targets and risk of common cancers: A mendelian randomization analysis
Source: PLoS Med. 2022 Feb 3;19(2):e1003897. doi: 10.1371/journal.pmed.1003897 (PMC8812899; doi:10.1371/journal.pmed.1003897)
Supplement: S2 Methods — (DOCX) [file pmed.1003897.s020.docx]

**S2 Methods. Sensitivity analyses using eQTL data**

We searched GTEx V8 (<https://gtexportal.org/home/>), eQTLGen (<https://www.eqtlgen.org/>), and BarcUVa-Seq to examine whether SNPs used to instrument ADRB1 and NCC were also eQTLs for the genes expressing these targets in one or more individual tissues or in whole blood (*P* < 5.0 x 10^-5^) (**S9 Table**). For SNPs that were also eQTLs, we then examined whether the direction of the effect of the SNP on gene expression was consistent with the direction of the effect of the SNP on SBP (harmonised to the SBP-lowering allele). Of 6 SNPs that were eQTLs for *ADRB1*, we removed 2 SNPs that influenced gene expression in the direction opposite to that expected based on the SBP lowering effect of the SNP (rs11196549, rs17875473) and removed one SNP where there was inconsistency in the direction of the effect of *ADRB1* expression across tissues (rs4918889). We then re-ran instrument validation and cancer endpoint Mendelian randomization analyses restricting instruments to 3 SNPs with evidence of being eQTLs that influenced gene expression levels in the direction expected given the direction of their effect on SBP levels (rs1801253, rs143854972, rs10787510) (**S10 Table**). We did not find evidence that the SNP used to instrument NCC influenced expression of the gene encoding this target.
