# Supplementary material for: Genetically proxied therapeutic inhibition of antihypertensive drug targets and risk of common cancers: A mendelian randomization analysis
Source: PLoS Med. 2022 Feb 3;19(2):e1003897. doi: 10.1371/journal.pmed.1003897 (PMC8812899; doi:10.1371/journal.pmed.1003897)
Supplement: S3 Methods — (DOCX) [file pmed.1003897.s021.docx]

**S3 Methods. BarcUVa-Seq study**

Individuals included in the BarcUVa-Seq study were participants of a Spanish colorectal cancer risk screening programme who obtained a normal colonoscopy result (i.e. macroscopically normal colon tissue, with no malignant lesions). RNA-Seq was performed using biopsy data collected from the superficial epithelial mucosa at colonoscopy and DNA genotyping was performed from leukocytes of peripheral blood samples. Biopsies were obtained from the ascending (31%), transverse (32%), and descending (37%) colon. Briefly, quality control of the sequencing reads was performed using FastQC, read alignment was performed using STAR, and gene expression quantification was performed using RSEM[1-3]. For downstream analyses, TMMs were computed from counts using only genes with more than 6 counts in at least 10 samples. Genotype imputation of the SNP array was performed with Minimac 4 using the TOPMed reference panel[4-6]. We only kept SNPs with high imputation quality (R^2^ > 0.7). RNA-Seq sequencing, quality control and analysis were performed as described elsewhere[7] .

**References**

1. Andrews S. FastQC: a quality control tool for high throughput sequence data (2010 [Available from: <http://www.bioinformatics.babraham.ac.uk/projects/fastqc>.

2. Dobin A, Davis CA, Schlesinger F, Drenkow J, Zaleski C, Jha S, et al. STAR: ultrafast universal RNA-seq aligner. Bioinformatics. 2013;29(1):15-21.

3. Li B, Dewey CN. RSEM: accurate transcript quantification from RNA-Seq data with or without a reference genome. BMC Bioinformatics. 2011;12:323.

4. Das S, Forer L, Schönherr S, Sidore C, Locke AE, Kwong A, et al. Next-generation genotype imputation service and methods. Nat Genet. 2016;48(10):1284-7.

5. Fuchsberger C, Abecasis GR, Hinds DA. minimac2: faster genotype imputation. Bioinformatics. 2015;31(5):782-4.

6. Taliun D, Harris DN, Kessler MD, Carlson J, Szpiech ZA, Torres R, et al. Sequencing of 53,831 diverse genomes from the NHLBI TOPMed Program. bioRxiv. 2019:563866.

7. Díez-Obrero Vea. Genetic effects on transcriptome profiles in colon epithelium provide functional insights for genetic risk loci. Cell Mol Gastroenterol Hepatol. 2021;In Press.
