# Supplementary material for: Genetically proxied therapeutic inhibition of antihypertensive drug targets and risk of common cancers: A mendelian randomization analysis
Source: PLoS Med. 2022 Feb 3;19(2):e1003897. doi: 10.1371/journal.pmed.1003897 (PMC8812899; doi:10.1371/journal.pmed.1003897)
Supplement: S4 Methods — (DOCX) [file pmed.1003897.s022.docx]

**S4 Methods. Co-expression network and gene-set enrichment analyses**

Gene co-expression network analysis was conducted with the WGCNA R package[1]. To calculate the adjacency matrix, we selected the soft-thresholding power of 8 to approximate scale-free topology. We transformed the adjacency into a topological overlap matrix and performed hierarchical clustering of genes and dynamic tree cut before assigning genes to modules (i.e. clusters of genes with similar expression patterns, assigned arbitrary colour codes). To decrease the number of genes per module, we did not merge modules whose expression profiles were very similar. We performed correlation analysis between the summary profile (eigengene) of each module and the ACE wGRS.

Genes associated with the ACE wGRS at *P* < 5.0 x 10^-3^, as well as genes located in the black module of the co-expression network, were further examined in gene-set enrichment analyses using FUMA gene2func. This tool implements a hypergeometric test of enrichment in a wide array of curated gene-sets from various sources, such as the Molecular Signatures Database (MsigDB)[2].

**References**

1. Langfelder P, Horvath S. WGCNA: an R package for weighted correlation network analysis. BMC Bioinformatics. 2008;9:559.

2. Watanabe K, Taskesen E, van Bochoven A, Posthuma D. Functional mapping and annotation of genetic associations with FUMA. Nat Commun. 2017;8(1):1826.
