# Supplementary material for: Genetically proxied therapeutic inhibition of antihypertensive drug targets and risk of common cancers: A mendelian randomization analysis
Source: PLoS Med. 2022 Feb 3;19(2):e1003897. doi: 10.1371/journal.pmed.1003897 (PMC8812899; doi:10.1371/journal.pmed.1003897)
Supplement: Attachment — Submitted filename: ACE Responses to peer review comments_09.12.21.docx [file pmed.1003897.s024.docx]

Requests from Editors:

1. Data availability statement: Please also provide weblinks or contact information to request the summary genetic association data from BarcUVa-Seq, Finngen consortium, Asia Colorectal Cancer Consortium and the Korean National Cancer Center CRC Study.

Response: Done.

2. Abstract: Conclusions: Line 30-32: We suggest revising to: “In this study, we observed that genetically-proxied long-term ACE inhibition was associated with an increased risk of colorectal cancer, warranting comprehensive evaluation of the safety profiles of ACE inhibitors in clinical trials with adequate follow-up.”

Response: Done.

3. Author summary: Why was this study done? We suggest tempering the second point, as there are also studies suggesting the opposite direction of association: “Conflicting epidemiological evidence suggests that long-term use of these medications could increase risk of cancer.”

Response: We have now revised this second point as follows:

“Some epidemiological studies have suggested that long-term use of these medications can increase cancer risk, though findings have been conflicting”

4. Author summary: What do these findings mean? “These findings provide support for a link between long-term inhibition of the drug target for ACE inhibitors and colorectal cancer risk, highlighting the need to evaluate the safety profiles of these medications in clinical trials with adequate follow-up length.”

Response: Done.

5. Author summary: What do these findings mean? We suggest an additional bullet point, perhaps putting the clinical interpretations into context given limitations of the study.

Response: We have now added in the following bullet point into the “What do these findings mean?” subsection of the Author summary:

“Prior to confirmation of an effect of ACE inhibitor use on colorectal cancer risk in clinical trials, these findings should not alter clinical practice for ACE inhibitor prescribing.”

6. Introduction: Line 82: Here and throughout, please do not include spaces within brackets where multiple references are listed [2,3]. Please include a space between the end of the preceding word and the bracket.

Response: Done.

7. Introduction: Line 107-109: The phrasing of this sentence seems to imply there is no evidence ot suggest a chemo-preventive effect on cancer risk for ACE inhibitors. It may be helpful to mention here and/or elsewhere in the introduction, studies that do seem to suggest such a relationship, for example: <https://doi.org/10.1161/HYPERTENSIONAHA.120.15317> and the meta analysis mentioned in the Discussion section [68]. “In contrast to ACE inhibitors, some in vitro and epidemiological studies have suggested potential chemo-preventive effects of these medications on cancer risk though findings have been inconclusive [20-28].”

Response: We have now mentioned that some epidemiological studies have reported inverse associations between ACE inhibitor use and cancer risk, citing the Cheung et al. 2020 manuscript and Chen et al. 2020 meta-analysis highlighted by the editors.

As can now be seen on pg 8 lines 88-89 (clean version of revised manuscript):

“Some observational epidemiological studies have suggested potential adverse effects of long-term use of these drugs on risk of common cancers (i.e. breast, colorectal, lung, and prostate) [7-10], though findings have been largely inconsistent (i.e. null and protective associations have also been reported for the relationship between ACE inhibitor use and cancer risk) [11-15].”

We have also removed “In contrast” from this sentence starting with “In contrast to ACE inhibitors,…” and have rephrased as follows to emphasise that findings for beta blockers and thiazide diuretics, as with those for ACE inhibitors, have been inconsistent (pg 9 lines 108-110):

“Some in vitro and epidemiological studies have suggested potential chemo-preventive effects of these medications on cancer risk though findings have been inconclusive [22-30].”

8. Methods: Line 234: Please reference the specific section of the Supporting Information where this description can be found.

Response: We have revised this line as follows:

“An extended description of colocalisation analysis including assumptions of this method is presented in **Supplementary Material** (**Supplementary Methods** section).”

9. Methods: Line 254-256: It would be helpful to clarify here when analyses in the results are described as “weak evidence” to support associations. Thank you for your explanation of the description of results as “weak evidence” in your response letter. In the analyses section of the methods, please explain the definition that you use to classify evidence as “weak” or not. “For this reason, we would prefer to describe, for example, the strength of evidence for our findings for ACE inhibition and stroke (P=0.06) and coronary artery disease (P=0.16) as “weak evidence”, as advocated elsewhere (Kirkwood BR & Sterne JAC. Essential medical statistics (2nd edn). Blackwell Science, Oxford, 2003).” For example, at Line 321: Please clarify if this meets the Bonferroni-corrected threshold, or if this would be characterized as weak evidence, for the association with distal colon cancer: “ (OR 1.15, 95% CI 1.03-1.27; P = 0.01).” Please also clarify this at lines 344-345 “(OR equivalent
to 1 mmHg lower SBP: 0.87, 95% CI 0.79-0.96; P = 0.008)” and Line 356: “(OR equivalent to 1 mmHg lower SBP: 1.20, 95% CI 1.02-1.40; P = 0.03).” and Line 364: “(1.40, 95% CI 1.02-1.92; P = 0.035).” and Line 385: “(OR 1.13, 95% CI: 0.96-1.32; P = 0.14)”

Response: We have now clarified the *P*-value thresholds used as a heuristic to classify evidence as “strong” and “weak” across primary drug target analyses (i.e. not applied to “replication analysis in Europeans and exploratory analyses in East Asians” or MR follow-up of Bonferroni-significant transcriptome-wide analyses adjusting for expression of relevant genes). As can now be seen on pg 14 lines 255-258:

“To account for multiple testing across primary drug target analyses, a Bonferroni correction was used to establish a *P*-value threshold of < 0.0014 (false positive rate = 0.05/36 statistical tests [3 drug targets tested against 12 cancer endpoints]), which we used as a heuristic to define “strong evidence”, with findings between *P* ≥ 0.0014 and *P* < 0.20 defined as “weak evidence”. “

We have clarified that evidence for association was “weak” in the primary drug target MR passages highlighted by the Editor(s).

10. Methods: Line 285-286: Please provide a reference for the STROBE-MR.

Response: Done.

11. Results: Line 337-338: If possible, please also describe the main results from the analyses with the other cancer types in the text.

Response: We have now altered this passage as follows (pg 18 lines 340-344):

“There was little evidence that genetically-proxied ACE inhibition was associated with risk of breast cancer (OR 0.98, 95% CI 0.94-1.02; *P* = 0.35) or lung cancer (OR 1.01, 95% CI 0.92-1.10; *P* = 0.93) and weak evidence for an association with prostate cancer risk (OR 1.06, 95% CI 0.99 to 1.13; *P* = 0.08). Likewise, there was little evidence of association of genetically-proxied ACE inhibition with these cancers in histological subtype-stratified analyses (**Table 2**).”

12. Results: Line 349-350: Please change “similar to” to “consistent with” in this sentence: “In sensitivity analyses restricting the ADRB1 instrument to SNPs that are eQTLs for ADRB1, findings were similar to those obtained when using the primary instrument for this target (S10 Table).”

Response: Done.

13. Results: Line 392-393: “ACE was in the black module along with another 659 genes.” Please clarify here or in the Methods briefly what is indicated by the “black module” as this appears to be the first mention in the text.

Response: In the Methods (pg 15 lines 281-284), we now emphasise that the colour code assigned to each module (i.e. cluster of genes with similar expression patterns) is arbitrary:

“… or permit the identification of clusters of genes (termed “modules” and assigned arbitrary colour codes) which show a coordinated expression pattern associated with the wGRS (co-expression network analysis).”

We have also reiterated that modules are assigned arbitrary colour codes in the “Co-expression network and gene-set enrichment analyses” subsection of the Supporting Information.

14. Conclusions: Line 537-538: We suggest revising to “Our Mendelian randomization analyses suggest that genetically-proxied long-term inhibition of ACE is associated with increased risk of colorectal cancer.”

Response: Done.

15. Conclusions: Line 539-541: We suggest revising to: “Our findings provide human genetic support to results from short-term randomised trials suggesting that long-term use of β blockers and thiazide diuretics may not influence risk of common cancers.” or similar.

Response: We have made the edit suggested by the Editor(s).

16. References: Please check each reference for formatting. Please use the "Vancouver" style for reference formatting, and see our website for other reference guidelines <https://journals.plos.org/plosmedicine/s/submission-guidelines#loc-references>

For example, in Ref 2 “The lancet oncology” should be “Lancet Oncol” and please remove the Competing Interest information from Ref 8, Ref 60, Ref 72, and Ref 82.

Response: Done.

17. Tables 2, 3, and 4: Please define all abbreviations in the legend (ER, OR, CI).

Response: Done.

18. Page 42: Please remove the section titled “Funding” and ensure all information is completely and accurately entered into the “Financial Disclosures” section of the manuscript submission system. For consortia funding information, it is not clear if this is intended as part of the Acknowledgements, but it may be preferable to move this information to a supporting information file.

Response: Done. We have moved consortia acknowledgments and funding to the Supporting Information file.

19. Page 53-54: Please remove the sections “Author contributions” “Competing Interests” and “Data and materials availability” from the main text, and please make sure all information is complete and accurate in the relevant sections of the manuscript submission system.

Response: Done.

20. Page 55: Figure legends: Please be sure that each figure legend fully describes all abbreviations used, and explains the meaning behind all points/lines included.

Response: Done.

21. STROBE checklist: Thank you for including the STROBE-MR checklist. Please submit the checklist as a table format. Please also update the reference (doi: 10.1001/jama.2021.18236).

Response: Done.

22. Supporting Information files: Please list the titles and captions for each Table/Figure/Text at the end of your manuscript file.

Response: Done.

23. Supporting Information Tables 11-14: We would suggest that each table be provided as an independent file rather than a tab within one file. Please list the captions for the Tables at the end of your manuscript file. You may include a caption within the supporting information file itself, as long as that caption is also provided in the manuscript file.

Response: Done (please note that these Tables are now labelled S13-16).
