# Supplementary material for: Genetically proxied therapeutic inhibition of antihypertensive drug targets and risk of common cancers: A mendelian randomization analysis
Source: PLoS Med. 2022 Feb 3;19(2):e1003897. doi: 10.1371/journal.pmed.1003897 (PMC8812899; doi:10.1371/journal.pmed.1003897)
Supplement: Attachment — Submitted filename: 19.08.21 Response to reviewer comments.docx [file pmed.1003897.s023.docx]

We would like to thank the editors and reviewers for their helpful comments which we have addressed, point-by-point, below:

Requests from the editors:

1. Title: Please revise your title according to PLOS Medicine's style. Your title must be nondeclarative and not a question. It should begin with main concept if possible. "Effect of" should be used only if causality can be inferred, i.e., for an RCT. Please place the study design ("A randomized controlled trial," "A retrospective study," "A modelling study," etc.) in the subtitle (ie, after a colon).

Response: We have now modified the title to: “Genetically-proxied therapeutic inhibition of antihypertensive drug targets and risk of common cancers: A Mendelian randomization analysis”

2. Authors: Please provide information on where the members of the International Lung Cancer Consortium are listed (in the acknowledgements or in a supporting information file).

Response: We now mention that information on members of the International Lung Cancer Consortium is presented in the Acknowledgements.

3. Abstract: Methods and Findings: We suggest including a short introductory sentence, where you present the overall the study design.

Response: As can be seen on pg 5 lines 6-8 in “19.08.21 ACE cancer Main Text_revisions tracked” we have now included a short introductory sentence presenting the overall study design at the start of the Abstract Methods and Findings section:

“We performed a Mendelian randomization analysis to examine the association between genetically-proxied inhibition of three antihypertensive drug targets and risk of four common cancers (breast, colorectal, lung, and prostate).”

4. Abstract: Methods and Findings: Please note that the included GWAS data and FinnGen consortium data was limited to individuals of European ancestry. If space permits, it may be helpful to include/mention the analyses to see if findings could be extended to individuals of East Asian ancestry.

Response: As can be seen on pg 5 lines 15-16 we now mention that cancer GWAS and FinnGen were performed in individuals of European ancestry:

“Cancer GWAS and FinnGen consortia data were restricted to individuals of European ancestry.”

As we used a different approach to instrument construction for ACE inhibition in East Asians (i.e. different to that already described in the Abstract Methods and Findings section for Europeans), we were concerned that there would not be sufficient space in this section to also describe and present results for East Asian analyses. If, however, the Editors insist on including information on analyses performed in East Asians, we could try to fit this into the abstract (while maintaining a 500 word maximum).

5. Author summary: At this stage, we ask that you include a short, non-technical Author Summary of your research to make findings accessible to a wide audience that includes both scientists and non-scientists. The Author Summary should immediately follow the Abstract in your revised manuscript. This text is subject to editorial change and should be distinct from the scientific abstract. Please see our author guidelines for more information: <https://journals.plos.org/plosmedicine/s/revising-your-manuscript#loc-author-summary>

Response: We have now included an Author summary of our work which follows the Abstract.

6. Throughout (including in the Supporting Information file): Please use square brackets for in-text citations, placed before the sentence punctuation. Where multiple references are indicated, please do not include spaces within brackets.

Response: Done.

7. Methods: Please ensure that the study is reported according to the STROBE guideline, and include the completed STROBE checklist as Supporting Information. Please add the following statement, or similar, to the Methods: "This study is reported as per the Strengthening the Reporting of Observational Studies in Epidemiology (STROBE) guideline (S1 Checklist)."

The STROBE guideline can be found here: <http://www.equator-network.org/reporting-guidelines/strobe/>

When completing the checklist, please use section and paragraph numbers, rather than page numbers.

Response: Done. We used the checklist from the STROBE-MR guidelines preprint (https://peerj.com/preprints/27857/) as many of the reporting items are of more relevance to our MR analysis than reporting items included in STROBE documents for conventional observational epidemiological studies. If the Editors insist that we use a STROBE reporting checklist for conventional observational studies, this can also be accommodated.

On pg 16 lines 290-292 we state that:

"This study is reported as per the Guidelines for strengthening the reporting of Mendelian randomization studies (STROBE-MR) checklist (S1 Checklist).”

8. Methods: Did your study have a prospective protocol or analysis plan? Please state this (either way) early in the Methods section.

Response: On pg 16 lines 288-290 we have now stated that: “There was no formal prespecified protocol for this study. All analyses described above were decided a priori except those designated as “post-hoc” where additional sensitivity analyses were performed in response to peer review comments.”

a) If a prospective analysis plan (from your funding proposal, IRB or other ethics committee submission, study protocol, or other planning document written before analyzing the data) was used in designing the study, please include the relevant prospectively written document with your revised manuscript as a Supporting Information file to be published alongside your study, and cite it in the Methods section. A legend for this file should be included at the end of your manuscript.

Response: N/A.

b) If no such document exists, please make sure that the Methods section transparently describes when analyses were planned, and when/why any data-driven changes to analyses took place.

Response: As mentioned above, we now state on pg 16 lines 288-290 that: “There was no formal prespecified protocol for this study. All analyses described above were decided a priori except those designated as “post-hoc” where additional sensitivity analyses were performed in response to peer review comments.”

c) In either case, changes in the analysis-- including those made in response to peer review comments-- should be identified as such in the Methods section of the paper, with rationale.

Response: We now state and provide rationale for post-hoc analyses (i.e. those made in response to peer review comments) introduced into the analysis on pg 12 lines 191-194 and pg 14 lines 250-255.

9. Results: Line 255: Please clarify if “weak evidence” is intended to indicate that the result did not reach statistical significance (and if so, please clarify in the text). Similarly, please clarify this throughout the results (e.g. “little evidence” at line 259 and elsewhere).

Response: While we acknowledge conventional use of a 5% false positive threshold to define “statistical significance”, in line with recommendations from the American Statistical Association for the reporting and interpretation of P-values (Wasserstein RL & Lazar NA. The ASA's statement on p-values: context, process, and purpose. Am. Stat. 70, 129–133. 2016) we believe that it is more informative to avoid classifying the strength of evidence against the null hypotheses tested (which is on a continuum) in a binary manner. For this reason, we would prefer to describe, for example, the strength of evidence for our findings for ACE inhibition and stroke (P=0.06) and coronary artery disease (P=0.16) as “weak evidence”, as advocated elsewhere (Kirkwood BR & Sterne JAC. Essential medical statistics (2nd edn). Blackwell Science, Oxford, 2003).

10. Discussion: Lines 392-395: If relevant, it would be interesting to expand slightly on the discussion of aspirin’s protective effects and how this fits with your findings regarding ACE inhibition.

Response: In the passage describing the protective effects of long-term aspirin use on colorectal cancer risk in clinical trials, we now emphasise that these findings illustrate how long induction periods for development of colorectal cancer may mean that potential carcinogenic effects of the use of particular medications (e.g. ACE inhibitors) may not emerge until many years after initiation of pharmacological treatment.

On pg 22 lines 450-451 we now state the following:

“It is therefore possible that adverse effects of ACE inhibition on colorectal cancer risk may likewise not emerge until many years after treatment initiation.”

11. Discussion: Although you mention this in the Limitations paragraph, please consider if it would be helpful to expand on the discussion/implications of the apparent absence of evidence supporting an association between genetically-proxied ACE inhibition and colorectal cancer risk in the East Asian cohort.
Response: We have now elaborated further on the need to replicate ACE and colorectal cancer analyses with stronger instruments for ACE (currently limited to a single SNP with relatively lower instrument strength than that used for the European ACE instrument) to examine whether lack of transportability of findings across ancestries reflects low power in East Asian analyses or differences in local LD structure across ancestries. As can now be seen on pg 23 lines 488-491:

“Identification of stronger genetic instruments for ACE inhibition in East Asian populations can help to uncover whether the lack of transportability of ACE and colorectal cancer findings from Europeans to East Asians reflects lower statistical power in the latter or differences in local LD structure across ancestries.”

12. References: Please ensure the use of "Vancouver" style for reference formatting (For example, please update the information in reference 62), and see our website for other reference guidelines <https://journals.plos.org/plosmedicine/s/submission-guidelines#loc-references>

Response: Done.

13. Figures 1-8: If possible, please provide descriptive legends for these, facilitating interpretation by a non-specialist audience.

Response: We now provide descriptive legends for Figures 1-8 on pg 56 of the Main text.

14. Supporting information tables S11-S14: Please provide descriptive legends in addition to titles for each individual table in the file.

Response: In addition to titles for Supporting information tables S11-S14, we now also provide descriptive legends for each of these tables as a separate “Descriptive legends for tables” tab in the “19.08.21 ACE cancer Tables S11-14 (revised).xlsx” file.


Comments from the reviewers:

Reviewer #1:

1. Not all ACE inhibitors are the same. The particular agents used in practice vary considerably in their pharmacological properties - both pharmacodynamic and pharmacokinetic. The authors completely overlook this, but it is critical to the potential future clinical investigation of their findings. This should be discussed in the limitations section. Do currently licensed ACE inhibitor have pharmacological effects in the colon?

Response: We’d like to thank the reviewer for highlighting this important point. In the Discussion, we now emphasise that different ACE inhibitors available on the market can differ in their pharmacological properties which has implications for future assessment of the potential carcinogenic role of long-term pharmacological inhibition of ACE.

As can now be seen on pg 25 line 519-525 of the revised Main text:

“There are different types and classes of ACE inhibitors, which vary in their pharmacodynamic and pharmacokinetic properties[83]. These differing pharmacological properties (e.g. differential absorption rate, affinity for tissue-bound ACE, and plasma half-life) can influence therapeutic benefit (or experience of adverse effects) of ACE inhibitors[84, 85]. Future evaluation of the potential effects of long-term ACE inhibitor use on cancer risk should therefore include assessment of whether findings are specific to individual agents or classes of ACE inhibitors.”

There is some evidence that currently licensed ACE inhibitors (e.g. captopril, enalapril, lisinopril ) have pharmacological effects in colon tissue in rodents (e.g. <https://pubmed.ncbi.nlm.nih.gov/15908024/>, <https://link.springer.com/article/10.1007%2Fs10620-013-2825-4>, <https://link.springer.com/article/10.1007%2Fs10620-006-9124-2>)

2. Page 11 line 203 onwards, what the authors describe is mediation, not "horizontal pleiotropy" (a term that I despise). If the authors insist on using such ambiguous and unintuitive terms, then it would be nice for them to at least get it right. Sorry, rant over.

Response: We have now clarified that an association between an instrument for a drug target and a previously reported risk factor for a cancer endpoint could reflect either vertical pleiotropy (i.e. “mediation”) or horizontal pleiotropy (i.e. a direct effect of the instrument on an outcome of interest)

As can now be seen on pg 14 lines 239-245 (note: “horizontal pleiotropy” was defined previous to this passage):

“If there was evidence for an association between a genetically-proxied drug target and previously reported risk factor (*P* < 0.05), this could reflect vertical pleiotropy (i.e. “mediated pleiotropy” where an instrument has an effect on two or more traits that influence an outcome via the same biological pathway) or horizontal pleiotropy. In the presence of an association with a previously reported risk factor multivariable Mendelian randomization can then be used to examine the association of drug target inhibition in relation to cancer risk, accounting for this risk factor[64].”

3. The authors talk about "additional assumptions of linearity" in both the abstract and discussion. I don't believe this is really that relevant. The purpose of this work is to identify a potential adverse effect of this drug class, not to estimate its magnitude in clinical practice. I'm sure the authors will themselves agree that they don't even attempt to do the latter. I don't believe these "additional assumptions of linearity" warrant nearly as much attention as the authors give them.

Response: We agree with the reviewer that the main emphasis of our analysis was the identification of a causal relationship between drug target inhibition and cancer risk, and not the magnitude of the effect estimate per se. We have removed mention of “non-linearity” from the “primary limitations” passage of the Abstract but have kept mention of the “assumption of linearity” in the Limitations section of the Discussion as effect estimates presented, to be interpretable, require an assumption of linearity between target-mediated SBP lowering and cancer risk.

4. The authors overlook the notable limitations of colocalization analysis, particularly false negative results and limited statistical power. These should be discussed and acknowledged as a limitation of the current work. It looks to me like there may be something there for SLC12A3 and ER -ve breast cancer, and more caution may be warranted in dismissing this.

Response: We agree with the reviewer that colocalisation analysis can have low statistical power which can lead to false negative findings. We now emphasise the low statistical power of this approach in the Limitations section.

As can now be seen on pg 23 lines 482-487:

“In addition, statistical power can be limited in colocalisation analysis which can reduce the probability of shared causal variants across traits examined being detected (i.e. leading to “false negative” findings). We therefore cannot rule out the possibility that some colocalisation findings suggesting low posterior probabilities of shared causal variants across traits (e.g. colocalisation analyses for genetically-proxied NCC inhibition and ER- breast cancer risk), reflected the limited power of this approach.”


Reviewer #2:

Re instrument selection, how do the instruments compare with previous similar work on other phenotypes by Gill et al Circulation 2019? The authors there only had one SNP for ACE inhibitors and was surprised to see such a difference. Also, why did you not include in this work CCBs? Did you think of improving the instruments limiting to eQTL expression?

Response: We’d like to thank the reviewer for their helpful comments. We used a different approach to instrument construction to that employed by Gill et al (Circulation 2019;140:270–279) who developed instruments for ACE inhibitors by selecting SNPs in *ACE* associated with systolic blood pressure (SBP) at genome-wide significance (resulting in 1 SNP). In our analyses we compared two different approaches to instrument construction for ACE inhibition: i) selecting SNPs in ACE associated with SBP at genome-wide significance in an updated GWAS meta-analysis of this trait (i.e. a similar approach to that used in Gill et al) which resulted in 2 SNPs and ii) selecting SNPs in ACE associated with serum ACE concentrations at genome-wide significance (resulting in 14 SNPs). In comparing the relative instrument strength across both approaches, we found that the “serum ACE concentrations” instrument was stronger than the “SBP SNPs in ACE” instrument (i.e. “serum ACE concentrations” instrument: r^2^ = 0.34-0.39, F= 2,156.5-2,594.9; “SBP SNPs in ACE” instrument: r^2^ =0.02, F=128.5) so we prioritised the former instrument as our primary instrument.

We opted not to include CCBs in this analysis because current instrument strategies are restricted to instrumenting different calcium channel subunits (i.e. there is no one single target for these medications, complicating inference and interpretation of findings). We wanted to restrict our analyses to medications with a single known target (i.e. ACE inhibitors, beta blockers, and thiazide diuretics) to permit clearer inferences of findings to be made.

Unlike for the ACE inhibition instrument where serum ACE concentrations data from a recent GWAS (Pigeyre et al. Diabetes Care 2020 Feb; dc191973) permitted us to develop instruments using pQTLs, we were not aware of any GWAS data with measures of ADRB1 or NCC available to develop pQTL instruments for these targets. In lieu of pQTL data for these two targets, we decided not to filter our SNPs further based on whether they were eQTLs for these genes because of potential poor overlap between strong eQTLs and pQTLs as documented elsewhere: <https://www.nature.com/articles/s41586-018-0175-2> , <https://www.ncbi.nlm.nih.gov/pmc/articles/PMC4507520/> , <https://www.nature.com/articles/s41576-020-0258-4> (i.e. potentially leading to the removal of SNPs that are strong pQTLs but poor eQTLs or enriching an instrument for SNPs that are strong eQTLs but not necessarily pQTLs). In internal analyses, for example, we found that only 4 of 14 pQTLs used to instrument serum ACE concentrations were also ACE eQTLs (in any tissue) in GTEx V8, eQTLGen or BarcUVa-Seq (2 SNPs were not available in GTEx). As an additional sensitivity analysis we have now i) examined whether our ADRB1 and NCC SNPs are also eQTLs for these genes (in any tissue or whole blood), and ii) re-ran MR analyses (target validation and cancer-endpoint) using only SNPs that showed some evidence of being an eQTL for the gene of interest.

We have now incorporated these additional sensitivity analyses into the manuscript. As can now be seen on pg 14 lines 250-255 (Main text):

“As an additional post-hoc sensitivity analysis we also evaluated whether SNPs used to instrument ADRB1 and NCC inhibition were also eQTLs (expression quantitative trait loci) for the genes encoding these proteins. Instrument validation and cancer endpoint Mendelian randomization analyses were then repeated by restricting instruments to SNPs showing evidence of being eQTLs for these targets (Additional information on these sensitivity analyses is provided in **Supplementary Material**).”

We now state on pg 3 of **Supplementary Material**:

“We searched GTEx V8 (<https://gtexportal.org/home/>), eQTLGen (<https://www.eqtlgen.org/>), and BarcUVa-Seq to examine whether SNPs used to instrument ADRB1 and NCC were also eQTLs for the genes expressing these targets in one or more individual tissues or in whole blood (*P* < 5.0 x 10^-5^) (**S9 Table**). For SNPs that were also eQTLs, we then examined whether the direction of the effect of the SNP on gene expression was consistent with the direction of the effect of the SNP on SBP (harmonised to the SBP-lowering allele). Of 6 SNPs that were eQTLs for *ADRB1*, we removed 2 SNPs that influenced gene expression in the direction opposite to that expected based on the SBP lowering effect of the SNP (rs11196549, rs17875473) and removed one SNP where there was inconsistency in the direction of the effect of *ADRB1* expression across tissues (rs4918889). We then re-ran instrument validation and cancer endpoint Mendelian randomization analyses restricting instruments to 3 SNPs with evidence of being eQTLs that influenced gene expression levels in the direction expected given the direction of their effect on SBP levels (rs1801253, rs143854972, rs10787510) (**S10 Table**). We did not find evidence that the SNP used to instrument NCC influenced expression of the gene encoding this target.”

In the Results of the **Main text** (pg 18 lines 355-356) we now state:

“In sensitivity analyses restricting the ADRB1 instrument to SNPs that are eQTLs for ADRB1, findings were similar to those obtained when using the primary instrument for this target (**S10 Table**).”

We now also mention in the Limitations section that we did not find evidence that the SNP used to instrument NCC was also an eQTL for this gene (pg 24 lines 496-499):

“Third, while we were able to perform sensitivity analyses for ADRB1 inhibition by restricting instruments to SNPs that were also eQTLs for ADRB1, we were unable to find evidence that the SNP used to instrument NCC (rs35797045) was also an eQTL for the gene encoding this target.”

How do you explain the lack of association with stroke?

Response: As the reviewer notes, though we found that our ADRB1 instrument was associated with lower risk of coronary artery disease (consistent with findings from meta-analyses of RCTs of beta blockers), we did not find clear evidence for an association with stroke. It is plausible that this finding could reflect a potential lower prevalence of antihypertensive medication among those with a genetic predisposition to lower SBP mediated via ADRB1 levels in the MEGASTROKE consortium dataset that we obtained summary data for stroke risk from. For example, we found that the G allele for the top SNP used to proxy ADRB1 (rs1801253) associates with both lower SBP and lower odds of “taking blood pressure medication” in the UK Biobank (<https://gwas.mrcieu.ac.uk/datasets/ukb-b-18009/>). Though an attenuation of an effect (if systematic across the instrument) would bias both CAD and stroke estimates, if the original CAD estimate were stronger in magnitude, this could account for the instrumenting still showing an association with CAD risk. It is also obviously possible that this association between ADRB1 and stroke risk could simply reflect the presence of horizontal pleiotropy biasing the effect estimate toward the null and/or a non-target-mediated effect of these medications.

We now mention again the Limitations that we were only able to recapitulate known beta blocker effects using our ADRB1 instrument for one of two positive control analyses and discuss potential reasons for this. As can be seen on pg 24 lines 502-507:

“Likewise, in “instrument validation” analyses for ADRB1, our inability to recapitulate known effects of ADRB1 inhibition (via beta blockers) on stroke risk could reflect the aforementioned inability to account for blood pressure medication users in the stroke datasets analysed. It is also possible that these findings reflect the presence of horizontal pleiotropy in the instrument biasing the effect estimate toward the null and/or a non-target-mediated effect of these medications on stroke risk.”

Could the associations just reflect associations between BP levels and phenotypes as these are BP SNPs? Gill et al PheWAS on the same drugs has also cancer outcomes with null associations including ACE and colon cancer. Did you try to replicate your findings in UK Biobank available data?

Response: When we examined the relationship between genetically-proxied systolic blood pressure (i.e. constructing an instrument from genome-wide significant SBP SNPs agnostic of genomic position), we found little evidence of an association suggesting that findings for ACE and colorectal cancer were not being driven through SBP per se. This is further supported by the null associations between genetically-proxied ACE inhibition and risk of the other two antihypertensive targets examined.

In the analysis of genetically-proxied ACE inhibition and colon cancer risk by Gill et al., there was likely less statistical power than ours to detect an effect given that the instrument was restricted to a single SNP in ACE (rs4291, r^2^:0.00037 vs 14 SNPs, r^2^:0.34-0.39 in our analysis) and analyses were restricted to 3,122 cases in UK Biobank (vs 32,002 colon cancer cases in our GECCO analysis).

The colorectal cancer data that we obtained from the GECCO consortium includes UK Biobank data already so we were not able to independently replicate this finding in this dataset. Our replication analyses were therefore performed in the FinnGen consortium.

Observational data on BP and colorectal cancer has shown positive associations - see Seretis et al Sci Rep 2019 on latest meta-analysis. How do you explain this in contacts of the ACE drug inhibition?

Response: We would like to thank the reviewer for pointing us to this meta-analysis of observational studies examining the association of hypertension and blood pressure with cancer risk. Findings from this meta-analysis suggest that hypertension (but not SBP) is associated with colorectal cancer risk. These findings could be consistent with our results as a large proportion of individuals with clinically-diagnosed hypertension are likely to be on anti-hypertensive medications, including ACE inhibitors (e.g. 49.5% of initiators of antihypertensive medications in a large UK primary care analysis were on combined ACE inhibitors/angiotensin receptor blockers: https://www.bmj.com/content/371/bmj.m4080). The absence of an association of SBP with colorectal cancer is consistent with our MR analyses that genetically-proxied SBP is unlikely to influence colorectal cancer risk (pg 18 lines 336-339).

A cautious statement on the continuous safety of these drugs is needed to avoid lack of adherence of the medications given these possible side effects until more data accumulates.

Response: We agree with the reviewer and have attempted to further emphasise that these findings are tentative and require confirmatory evidence (from long-term follow up of clinical trials) in order to guide clinical decision-making.

As can now be seen on pg 23 lines 479-482:

“Despite the evidence suggesting a link between genetically-proxied ACE inhibition and colorectal cancer, however, these findings cannot demonstrate a causal relationship between ACE inhibitor use and colorectal cancer risk; only a randomised controlled trial could establish this relationship.”


Reviewer #3:

        1. Selection of IVs for antihypertensive medications using GWAS of SBP is based on the assumption that the selected genetic variants affect SPB through changing function/level of antihypertensive drug targets. Can the authors demonstrate that the SBP GWAS SNPs they selected as IV s for ADRB1 and SLC12A3 can affect serum concentration of the corresponding proteins? If this information is not available  (no GWAS or PWAS for corresponding proteins), the authors have an access to the gene expression data (BarcUVa-Seq study) and should be able to demonstrate if selected genetic variants are associated with the expression level of the corresponding genes.

Response: We did not have protein expression data available to develop instruments for ADRB1 and NCC so used a “downstream biomarker” drug-target MR approach to develop our instruments, as has been used previously (e.g. <https://www.nejm.org/doi/full/10.1056/nejmoa1604304> , <https://www.ahajournals.org/doi/suppl/10.1161/CIRCULATIONAHA.118.038814> , <https://pubmed.ncbi.nlm.nih.gov/28846118/> ) and advocated in reviews of drug-target MR (e.g. <https://wellcomeopenresearch.org/articles/6-16>). This approach assumes no direct effect of instruments on an outcome of interest (i.e. no effect of drug target SNPs on cancer risk other than that mediated through target modulation).

In lieu of pQTL data for ADRB1 and NCC, we opted not to filter our SNPs further based on whether they were eQTLs for these genes because of potential poor overlap between strong eQTLs and pQTLs as documented elsewhere: <https://www.nature.com/articles/s41586-018-0175-2> , <https://www.ncbi.nlm.nih.gov/pmc/articles/PMC4507520/> , <https://www.nature.com/articles/s41576-020-0258-4> (i.e. potentially leading to the removal of SNPs that are strong pQTLs but poor eQTLs or enriching an instrument for SNPs that are strong eQTLs but not necessarily pQTLs). In internal analyses, for example, we found that only 4 of 14 pQTLs used to instrument serum ACE concentrations were also ACE eQTLs (in any tissue) in GTEx V8, eQTLGen or BarcUVa-Seq (2 SNPs were not available in GTEx). As an additional sensitivity analysis we have now i) examined whether our ADRB1 and NCC SNPs are also eQTLs for these genes (in any tissue or whole blood), and ii) re-ran MR analyses (target validation and cancer-endpoint) using only SNPs that showed some evidence of being an eQTL for the gene of interest.

As can be seen in the Table below (also **Table S9**), using GTEx V8 (<https://gtexportal.org/home/>), eQTLGen (<https://www.eqtlgen.org/>), and BarcUVa-Seq, 6 of 8 ADRB1 SNPs are eQTLs for ADRB1 in individual tissues or in whole blood (*P* < 5.0 x 10^-5^). However for 2 of the 6 SNPs (rs11196549, rs17875473) the direction of expression was not consistent with the direction of effect on SBP and for 1 of the 6 SNPs (rs4918889), of the 3 tissues that this SNP was an eQTL for, 1 of the 3 was not in the direction consistent with the effect of this SNP on SBP. We did not find evidence to support the SNP used to instrument NCC influencing expression of the gene encoding this target.

| **Target** | **EA/**  **NEA** | **Effect (SE)**  **(SBP)** | ***P*-value**  **(SBP)** | **Tissue**  **(gene expression)** | **Beta**  **(gene expression)** | ***P*-value**  **(gene expression)** |
| --- | --- | --- | --- | --- | --- | --- |
| *ADRB1* |  |  |  |  |  |  |
| rs1801253 | G/C | -0.41 (0.03) | 8.07x10^-43^ | Testis | -0.46 (NES) | 3.5 x10^-13^ |
| rs11196549 | G/A | -0.62 (0.07) | 2.53x10^-19^ | Whole blood | 4.37  (Z) | 1.2 x10^-5^ |
| rs4918889 | G/C | -0.30 (0.04) | 7.53x10^-18^ | Whole blood | 18.43 (NES) | 7.0 x10^-76^ |
|  |  |  |  | Colon | -0.31  (TMM) | 4.8 x10^-5^ |
|  |  |  |  | Muscle (Skeletal) | -0.31 (NES) | 2.5 x10^-6^ |
| rs460718 | A/G | -0.24 (0.03) | 2.21x10^-17^ | - | - | - |
| rs11196597 | G/A | -0.27 (0.04) | 3.07x10^-12^ | - | - | - |
| rs143854972 | G/A | -0.39 (0.06) | 4.35x10^-11^ | Whole blood | -10.73 (Z) | 7.7 x10^-27^ |
| rs17875473 | C/T | -0.28 (0.05) | 9.04x10^-9^ | Whole blood | 6.3  (Z) | 2.7 x10^-10^ |
| rs10787510 | A/G | -0.15 (0.03) | 2.01x10^-8^ | Whole blood | -5.48 (Z) | 4.3 x10^-8^ |
|  |  |  |  | Pancreas | -0.31 (NES) | 2.0 x10^-5^ |
|  |  |  |  |  |  |  |
| *NCC* |  |  |  |  |  |  |
| rs35797045 | A/C | -0.35 (0.06) | 4.85x10^-8^ | - | - | - |

EA = Effect Allele, NEA = Non-Effect Allele, SE = Standard Error, NES = Normalized Effect Size (obtained from GTex V8), Z = Z-statistic (obtained from blood EQTL gen), TMM = Trimmed Mean of M-value (obtained from Barc-UVa-Seq).

When we re-ran our “instrument validation” analyses using only 3 SBP SNPs that showed consistent evidence of being an eQTL for ADRB1 in the direction consistent with blood pressure lowering (in any tissue), our findings did not differ markedly from analyses using all 8 ADRB1 SNPs:

| **Instrument** | **Endpoint** | **OR (95% CI)** | ***P*-value** |
| --- | --- | --- | --- |
| 8 SNP | CAD | 0.95 (0.92-0.98) | 1.5 x 10^-3^ |
| 3 SNP | CAD | 0.96 (0.91-1.00) | 0.06 |
| 8 SNP | Stroke | 1.03 (0.99-1.07) | 0.18 |
| 3 SNP | Stroke | 1.01 (0.96-1.06) | 0.68 |

Scaled to represent ADRB1 inhibition equivalent of a 1 mmHg SBP reduction

Likewise, when we re-ran our cancer endpoint analyses using these 3 SNPS, our findings did not markedly differ from analyses using 8 ADRB1 SNPs:

| **Outcome** | **OR (95% CI)** | ***P*-value** |
| --- | --- | --- |
| Breast cancer (8 SNP) | 1.01 (0.99-1.04) | 0.38 |
| Breast cancer (3 SNP) | 1.01 (0.98-1.04) | 0.56 |
| ER+ Breast cancer (8 SNP) | 1.01 (0.98-1.04) | 0.44 |
| ER+ Breast cancer (3 SNP) | 1.02 (0.99-1.06) | 0.24 |
| ER- Breast cancer (8 SNP) | 0.98 (0.94-1.02) | 0.38 |
| ER- Breast cancer (3 SNP) | 0.97 (0.91-1.03) | 0.29 |
|  |  |  |
| Colorectal cancer (8 SNP) | 0.98 (0.96-1.01) | 0.31 |
| Colorectal cancer (3 SNP) | 0.95 (0.91-1.00) | 0.04 |
| Colon cancer (8 SNP) | 0.99 (0.95-1.03) | 0.63 |
| Colon cancer (3 SNP) | 0.96 (0.91-1.02) | 0.18 |
| Rectal cancer (8 SNP) | 1.00 (0.95-1.04) | 0.84 |
| Rectal cancer (3 SNP) | 0.94 (0.88-1.01) | 0.09 |
|  |  |  |
| Lung cancer (8 SNP) | 1.01 (0.96-1.07) | 0.64 |
| Lung cancer (3 SNP) | 1.01 (0.94-1.09) | 0.73 |
| Lung adenocarcinoma (8 SNP) | 0.98 (0.91-1.04) | 0.48 |
| Lung adenocarcinoma (3 SNP) | 1.00 (0.92-1.09) | 0.94 |
| Small cell lung carcinoma (8 SNP) | 0.87 (0.79-0.96) | 0.008 |
| Small cell lung carcinoma (8 SNP) | 0.89 (0.77-1.03) | 0.13 |
| Squamous cell lung cancer (8 SNP) | 0.98 (0.91-1.06) | 0.67 |
| Squamous cell lung cancer (3 SNP) | 0.94 (0.85-1.03) | 0.16 |
|  |  |  |
| Prostate cancer (8 SNP) | 1.00 (0.96-1.03) | 0.73 |
| Prostate cancer (2 SNP)* | 1.05 (0.97-1.13) | 0.24 |
| Advanced prostate cancer (8 SNP) | 1.00 (0.94-1.06) | 0.97 |
| Advanced prostate cancer (3 SNP) | 1.02 (0.95-1.10) | 0.51 |

Scaled to represent ADRB1 inhibition equivalent of a 1 mmHg SBP reduction. * Neither rs1801253 (nor a high LD proxy) was available in PRACTICAL for prostate cancer risk.

We have now incorporated these additional sensitivity analyses into the manuscript. As can now be seen on pg 14 lines 250-255 (Main text):

“As an additional post-hoc sensitivity analysis we also evaluated whether SNPs used to instrument ADRB1 and NCC inhibition were also eQTLs (expression quantitative trait loci) for the genes encoding these proteins. Instrument validation and cancer endpoint Mendelian randomization analyses were then repeated by restricting instruments to SNPs showing evidence of being eQTLs for these targets (Additional information on these sensitivity analyses is provided in **Supplementary Material**).”

We now state on pg 3 of **Supplementary Material**:

“We searched GTEx V8 (<https://gtexportal.org/home/>), eQTLGen (<https://www.eqtlgen.org/>), and BarcUVa-Seq to examine whether SNPs used to instrument ADRB1 and NCC were also eQTLs for the genes expressing these targets in one or more individual tissues or in whole blood (*P* < 5.0 x 10^-5^) (**S9 Table**). For SNPs that were also eQTLs, we then examined whether the direction of the effect of the SNP on gene expression was consistent with the direction of the effect of the SNP on SBP (harmonised to the SBP-lowering allele). Of 6 SNPs that were eQTLs for *ADRB1*, we removed 2 SNPs that influenced gene expression in the direction opposite to that expected based on the SBP lowering effect of the SNP (rs11196549, rs17875473) and removed one SNP where there was inconsistency in the direction of the effect of *ADRB1* expression across tissues (rs4918889). We then re-ran instrument validation and cancer endpoint Mendelian randomization analyses restricting instruments to 3 SNPs with evidence of being eQTLs that influenced gene expression levels in the direction expected given the direction of their effect on SBP levels (rs1801253, rs143854972, rs10787510) (**S10 Table**). We did not find evidence that the SNP used to instrument NCC influenced expression of the gene encoding this target.”

In the Results of the **Main text** (pg 18 lines 355-356) we now state:

“In sensitivity analyses restricting the ADRB1 instrument to SNPs that are eQTLs for ADRB1, findings were similar to those obtained when using the primary instrument for this target (**S10 Table**).”

We now also mention in the Limitations section that we did not find evidence that the SNP used to instrument NCC was also an eQTL for this gene (pg 24 lines 496-499):

“Third, while we were able to perform sensitivity analyses for ADRB1 inhibition by restricting instruments to SNPs that were also eQTLs for ADRB1, we were unable to find evidence that the SNP used to instrument NCC (rs35797045) was also an eQTL for the gene encoding this target.”

        2. For the ACE IVs selection, the authors used two different datasets: (i)  a GWAS of SPB and  (ii) a GWAS of ACE concentration.  The authors prioritized an IV derived from the GWAS of serum ACE concentration. Though I agree with the selection (mostly due to the reason mentioned in the comment above), the authors reasoning behind IV selection is questionable. They compared proportion of variance in SPB and in ACE, explained by two IVs. The authors compared two different outcomes and, of course, they were not be the same. What if SBP has only limited genetic component to it and the current IV explained 100% of heritability with the r2=0.02, while serum ACE is completely driven by genetic and the current IV explains only small proportion of variance in SBP? I do not think those two measures are comparable (but please, prove me wrong if you think otherwise). I think it makes much more sense to compare two IVs using the same outcome. The authors can still derive two list of genetic variants from the GWAS of SBP and the GWAS of the serum ACE concentration and then compare the proportion of  the variance in the serum ACE concentration explained by two different instrumental variables (IV). The authors have an access to the summary level data from the serum ACE GWAS . So it should not be a problem.

Response: We agree with the reviewer that comparing both “serum ACE” and “SBP” instruments across the same outcome (i.e. serum ACE concentrations) is preferable to comparing these instruments across two different outcomes.

The two SNPs associated with SBP at genome-wide significance (*P*< 5 x 10^-8^) are rs8077276 and rs28656895 (pairwise r^2^= 0.059, CEU EUR). A comparison of instrument strength (in relation to serum ACE concentrations) for both instruments is below:

Comparison of instrument strength across instruments:

14 SNP ACE instrument: r^2^=0.34-0.39, F=2,156.5-2,594.9

2 SNP SBP instrument: r^2^=28.1%, F=759.9

We have now mentioned in the manuscript that we performed a post-hoc instrument selection step comparing r^2^/F-statistics for serum ACE concentrations across these two settings as a further step to prioritise the primary instrument for ACE inhibition. As can now be seen on pg 14 lines 191-194:

“As an additional instrument construction step, we also performed a post-hoc comparison of the proportion of variance in serum ACE concentrations explained by both instruments and found that the serum ACE concentrations instrument explained a larger proportion of the variance in this trait than the systolic blood pressure instrument (r^2^ = 0.28, F=759.9).”

        3. Going back to the IV selection. The author validated the IV by examining the association between stroke and coronary artery disease and comparing the direction of effects. Though I can understand authors way of thinking, wouldn't be easier to validate the IV by comparing it with the serum concentration of corresponding proteins or, at least, gene expression level of corresponding genes if proteomic data is not available? The observed associations with the risk of stroke or CAD could be due to multiple reasons (pleiotropy etc. ) and may have nothing to do with the inhibition of the corresponding proteins.

Response: As mentioned in our response to Question #1 from the reviewer, we would have preferred to develop our instrument against circulating protein levels of ADRB1 and NCC but, to the best of our knowledge, GWAS data on circulating levels of these proteins has not been published to date. We therefore used a “downstream biomarker” drug-target MR approach in this analysis.

As mentioned above, sensitivity analyses for ADRB1 using SNPs that are also eQTLs for ADRB1 (which influenced gene expression in the direction expected given their effect on SBP levels) were broadly consistent with findings from analyses using the primary ADRB1 instrument. We were not able to find evidence that the single SNP used to instrument NCC was also an eQTL which we have mentioned in the Limitations section of the Discussion.

The reviewer is correct that it is possible that SNPs in genes encoding drug targets that strongly associate with indications for the target (i.e. SBP) could still influence outcomes through horizontal pleiotropy. In the Limitations section we therefore explicitly state that we cannot rule out the possibility of our findings being driven by exchangeability or exclusion restriction violations. On pg 25 lines 534-537 we now also state that as GWAS data with circulating levels of ADRB1 and NCC become available there would be merit in evaluating whether findings presented in this analysis can be replicated when using alternate instruments developed from pQTLs for these targets.

“As data on circulating levels of ADRB1 and NCC become available in future GWAS, there would be merit in evaluating whether findings presented in this analysis can be replicated when using alternate instruments developed from protein quantitative loci for these targets.”

        4. To proxy ADRB1 inhibition, 8 SNPs associated with the SBP at genome-wide significance and in a weak LD with each other were selected. Have author considered using penalized regression for IV selection? It all sounds very arbitrary taking into account that there are multiple statistical methods available for feature selection .

Response: We thank the author for their suggestion to consider penalized regression for IV selection, a method that we were not familiar with. Our use of genome-wide significant SNPs in weak LD (r^2^<0.10) is a similar approach to that used by prior MR analyses examining drug targets. Though there is rationale for using this approach (e.g. use of genome-wide significant variants should minimise weak instrument bias across SNPs and use of weakly correlated SNPs can increase statistical power as documented elsewhere: https://www.nature.com/articles/s41467-020-16969-0), we agree with the reviewer that there is an element of arbitrariness in the particular P-value and LD parameters employed (i.e. these could be optimised through use of more advanced statistical methods for feature selection). Though likely out of the scope for this analysis, we thank the reviewer again for highlighting to us alternate models for IV selection which we will consider in our future work.

        5. Gene Expression analysis in colon is of particular interest as it has potential to show mechanism of action. However, it is not clear from the text if genetically proxied serum ACE inhibition was associated with low and high gene expression. Would it be possible to provide direction of effects for the gene expression results.

Response: We have now clarified the direction of effect of our wGRS for serum ACE concentrations on colon gene expression levels. As now emphasised in the text, there was an inverse association between the serum ACE concentrations wGRS and expression of colon ACE, CYB561, and FTSJ3. As mentioned in the text, we found little evidence for colocalisation of the colon ACE expression signal with colorectal cancer risk (including in updated colocalisation analyses paired with conditional analyses described below). As also mentioned in the text, *ACE*, *CYB561*, and *FTSJ3* are neighbouring genes on chromosome 17 suggesting that associations between the ACE wGRS and *CYB561* and *FTSJ3* could be driven through their co-expression. In multivariable MR analysis, we did not find evidence to suggest that an effect of genetically-proxied ACE inhibition was mediated via CYB561 expression (genetically-proxied FTSJ3 expression was not associated with colorectal cancer risk).

        6. Supplementary method. Colocalization analysis. The author made conclusions about multiple signals in the locus based on the inspection of locus zoom. I do not think one can make this conclusion without a formal conditional analysis of the locus (e.g conditional on the top variant).

Response: We have now incorporated conditional analyses using GCTA-COJO as further assessment of the presence of conditionally independent causal variants in colocalisation analysis. Briefly, we conditioned all SNPs in regions examined on the sentinel SNP (*P*<5 x 10^-8^) for each trait (where applicable) and then sequentially for each conditionally independent SNP (*P*< 5 x 10^-8^) in the region. Pairwise colocalisation analysis using “coloc” as implemented in PWCoCo (https://github.com/jwr-git/pwcoco) was then re-performed for ADRB1/small cell lung cancer, NCC/ER- breast cancer, and ACE colon expression/colorectal cancer risk using association statistics for SNPs conditioned on either the sentinel SNP (across either trait) or additional conditionally independent SNPs (across either trait). Pairwise colocalisation results using conditionally independent variants were similar to those using marginal statistics (i.e. top posterior probabilities for shared causal variants across the three comparisons ranged from 0.014-0.291)(**Tables S7, S11, S12**).

We noted an error in our code for colon ACE expression/colorectal cancer risk colocalisation analysis. Previously, the P4 (i.e. shared causal variant posterior probability) in colocalisation analysis using marginal statistics was provided as 0.076. In analyses using corrected code, P4 is now 0.291. (This has now been corrected in the text).

We have revised **Supplementary Methods** and **Results** sections to include mention of conditional analyses and colocalisation using conditional statistics.

        7. To test horizontal pleiotropy the authors examined associations between the Ivs for three drug targets and  possible risk factors. The selection of the possible risk factor is questionable. I think it should be expanded to alcohol, height and physical activity. Indeed some of the variants included as IV for ACE are associated with height as well, which as a known risk factor of CRC.

Response: We have now included assessment of the association between genetically-proxied ACE inhibition and alcohol consumption (measured as log-transformed “number of alcohol drinks per week” from a GWAS of 335,394 individuals of European ancestry; PMID: 30643251), standing height (N 461,950, IEU internal GWAS of UK Biobank), and physical activity (measured as “overall activity” from a GWAS of 91,105 participants, PMID: 30531941). We found little evidence of association across the three traits: (P=0.12 to 0.45). We have now updated **Table S5** to include these findings and mention looking at them in the Methods and Results.

        8. Finally, I know it may be speculative, but I think the discussion would benefit from calculating predicted . attributable fraction  . What is proportion of CRC incidence could be attributable to ACE inhibitors medication with a given proportion of population on ACE inhibitors.

Response: Given the somewhat novel nature of our findings, our preference would be not to calculate the proportion of CRC cases attributable to ACE inhibitors until additional confirmatory evidence of an effect of ACE inhibitors on cancer risk arises (preferably from confirmatory RCTs with long-term follow-up data). As highlighted by another reviewer of this manuscript, there is some concern of a potential lack of adherence to ACE inhibitors given the possible carcinogenic effects of these medications reported here. In line with this, in the Discussion of our manuscript, for example, we have presented a cautionary statement on the inability of our findings to establish a causal relationship between ACE inhibitor use and colorectal cancer risk (and the need for these findings to be replicated in clinical trial data). If the reviewer insists on us including this estimation, however, we would be able to accommodate this suggestion.

        Minor comments:
        1. The table S1 is confusing. It would be beneficial to provide more details how those estimates were generated and why they are different from the IV for ADRB1 provided in the table 1.  Would it be possible to show non adjusted estimates for all the variants presented in the table 1?  Why didn't you construct IV for SLC12A3 using GWAS unadjusted for BMI?

Response: Table S1 presents summary statistics for two SNPs used to instrument ADRB1 from a GWAS of systolic blood pressure that was not adjusted for body mass index or antihypertensive medication use (we have modified the title of this table to hopefully aid clarity). These estimates are different from Table 1 which presents summary statistics for ADRB1 SNPs from a larger GWAS meta-analysis of studies adjusted for body mass index and anti-hypertensive medication use used to develop our primary ADRB1 instrument. As stated in the Footnote to this table, there were no variants at *P* < 5 x 10^-8^ available to instrument NCC (SLC12A3) in analyses unadjusted for BMI.

        2. Please, bring all effect allele concordance across the manuscript. It will be easier for readers to follow direction of effects across different analysis. E.g for certain SNPs (e.g. rs12452187 and some other) table 1 shows one effect allele and table S2 shows opposite effect allele.

Response: We have now presented the same effect allele for all SNPs in the manuscript (we have also modified Table 2 to represent the same effect allele per SNP as those presented in Table 1 for consistency).

Table S5. Inhibition repeated twice . See comment above.  I suggest to add other established risk factors to the table, such as height, alcohol, physical activity ive

Response: We thank the reviewer for spotting this typo. We have now included height, alcohol, and physical activity as additional established risk factors into our analyses and this table.


Reviewer #4:

1.      I'm confused about what exactly "genetically proxied ACE / ADRB1 / NCC inhibition" refer to in the paper. For instance, based on my understanding,  "instruments to proxy ACE inhibition" are selected as the SNPs that are near the ACE gene region and are significantly associated with SBP (similarly for ADRB1 and NCC). However, I do not see the definition of genetically-proxied ACE inhabitation itself. Does it mean a combined association of these 14 SNPs on SBP, or on serum ACE concentration? On page 10 line 162, the paper says "we examined the association between genetically proxied ACE inhibition and i) systolic blood pressure, ii) risk of stroke …". When I read this sentence, it sounds like the "genetically proxied ACE inhibition" means the selected 14 SNPs themselves, otherwise I have no idea what is exactly calculated for the association between "genetically proxied ACE inhibition" and SBP.

Response: We thank the reviewer for this comment and apologise for any confusion in terms of what we refer to when mentioning “genetically proxied ACE/ADRB1/NCC inhibition”.

We used different methodological approaches to develop genetic instruments for ADRB1 and NCC inhibition as compared to ACE inhibition.

For ADRB1 and NCC inhibition, we pooled GWAS data from two SBP GWAS and then constructed instruments for these targets by obtaining SNPs associated with SBP at genome-wide significance (*P*<5 x 10^-8^) in the pooled analysis that were in or within 100kb from the gene encoding each respective target (permitting SNPs to be in weak LD, pairwise r^2^<0.10).

For ACE inhibition, we compared two approaches for instrumentation. The first approach was the same as that used to develop instruments for ADRB1 and NCC (i.e. we selected SNPs associated with SBP at genome-wide significance that were in or within 100kb from ACE that were permitted to be in weak LD, r^2^<0.10). Since we also had access to GWAS data on serum ACE concentrations from a previous GWAS, we also examined constructing an instrument to proxy ACE inhibition by selecting SNPs associated with serum ACE concentration at genome-wide significance that were in or within 100kb from ACE (again permitted to be in weak LD, r^2^<0.10).

We found that the second approach to instrumentation (i.e. selecting SNPs robustly associated with serum ACE concentrations) generated a stronger instrument (r^2^ = 0.34-0.39, F= 2,156.5-2,594.9) than the first approach (r^2^ =0.02, F=128.5) so prioritised this instrument as our primary instrument for ACE inhibition. As a sensitivity analysis, we also examined associations between an instrument constructed from genome-wide significant SBP SNPs (i.e. the first approach examined) and colorectal cancer risk.

We have now modified passages of the text when describing construction of the ACE instrument in order to enhance clarity regarding how these two potential ACE instruments were explored in addition to the specific ACE instrument used to examine “genetically-proxied ACE inhibition”. As can now be seen on pg 12 lines 183-190:

“We then compared the proportion of variance in either systolic blood pressure or serum ACE concentrations explained (r^2^) across each respective instrument to prioritise the primary instrument to proxy ACE inhibition. The “serum ACE concentrations instrument” (r^2^ = 0.34-0.39, F= 2,156.5-2,594.9) was prioritised as our primary instrument to examine genetically-proxied ACE inhibition because of stronger instrument strength as compared to the “systolic blood pressure instrument” (r^2^ =0.02, F=128.5). In sensitivity analyses we also examined the association between genetically-proxied ACE inhibition and cancer endpoints using the “systolic blood pressure” instrument.”

2.      I think the authors may need some more explanation and justification on selection of instruments to proxy ACE / ADRB1 / NCC inhibition. Why are SNPs that are near the ACE gene region and are significantly associated with SBP good instruments? Seems that the authors also mentioned GWAS for traits serum ACE concentration and ACE activity. Why not select instruments as SNPs that have are significant associated with these two traits? Why not select instruments as eQTLs of gene ACE expression?

Response: We used serum ACE concentrations to develop our instrument for ACE inhibition. We did not have protein expression data available to develop instruments for ADRB1 and NCC so used a “downstream biomarker” drug-target MR approach to develop our instruments, as has been used previously (e.g. <https://www.nejm.org/doi/full/10.1056/nejmoa1604304> , <https://www.ahajournals.org/doi/suppl/10.1161/CIRCULATIONAHA.118.038814> , <https://pubmed.ncbi.nlm.nih.gov/28846118/> ) and advocated in reviews of drug-target MR (e.g. <https://wellcomeopenresearch.org/articles/6-16>). This approach assumes no direct effect of instruments on an outcome of interest (i.e. no effect of drug target SNPs on cancer risk other than that mediated through target modulation).

In lieu of pQTL data for these two targets, we decided not to filter our SNPs further based on whether they were eQTLs for these genes because of potential poor overlap between strong eQTLs and pQTLs as documented elsewhere: <https://www.nature.com/articles/s41586-018-0175-2> , <https://www.ncbi.nlm.nih.gov/pmc/articles/PMC4507520/> , <https://www.nature.com/articles/s41576-020-0258-4> (i.e. potentially leading to the removal of SNPs that are strong pQTLs but poor eQTLs or enriching an instrument for SNPs that are strong eQTLs but not necessarily pQTLs). In internal analyses, for example, we found that only 4 of 14 pQTLs used to instrument serum ACE concentrations were also ACE eQTLs (in any tissue) in GTEx V8, eQTLGen or BarcUVa-Seq (2 SNPs were not available in GTEx). As an additional sensitivity analysis we have now i) examined whether our ADRB1 and NCC SNPs are also eQTLs for these genes (in any tissue or whole blood), and ii) re-ran MR analyses (target validation and cancer-endpoint) using only SNPs that showed some evidence of being an eQTL for the gene of interest.

We have now incorporated these additional sensitivity analyses into the manuscript. As can now be seen on pg 14 lines 250-255 (Main text):

“As an additional post-hoc sensitivity analysis we also evaluated whether SNPs used to instrument ADRB1 and NCC inhibition were also eQTLs (expression quantitative trait loci) for the genes encoding these proteins. Instrument validation and cancer endpoint Mendelian randomization analyses were then repeated by restricting instruments to SNPs showing evidence of being eQTLs for these targets (Additional information on these sensitivity analyses is provided in **Supplementary Material**).”

We now state on pg 3 of **Supplementary Material**:

“We searched GTEx V8 (<https://gtexportal.org/home/>), eQTLGen (<https://www.eqtlgen.org/>), and BarcUVa-Seq to examine whether SNPs used to instrument ADRB1 and NCC were also eQTLs for the genes expressing these targets in one or more individual tissues or in whole blood (*P* < 5.0 x 10^-5^) (**S9 Table**). For SNPs that were also eQTLs, we then examined whether the direction of the effect of the SNP on gene expression was consistent with the direction of the effect of the SNP on SBP (harmonised to the SBP-lowering allele). Of 6 SNPs that were eQTLs for *ADRB1*, we removed 2 SNPs that influenced gene expression in the direction opposite to that expected based on the SBP lowering effect of the SNP (rs11196549, rs17875473) and removed one SNP where there was inconsistency in the direction of the effect of *ADRB1* expression across tissues (rs4918889). We then re-ran instrument validation and cancer endpoint Mendelian randomization analyses restricting instruments to 3 SNPs with evidence of being eQTLs that influenced gene expression levels in the direction expected given the direction of their effect on SBP levels (rs1801253, rs143854972, rs10787510) (**S10 Table**). We did not find evidence that the SNP used to instrument NCC influenced expression of the gene encoding this target.”

In the Results of the **Main text** (pg 18 lines 355-356) we now state:

“In sensitivity analyses restricting the ADRB1 instrument to SNPs that are eQTLs for ADRB1, findings were similar to those obtained when using the primary instrument for this target (**S10 Table**).”

We now also mention in the Limitations section that we did not find evidence that the SNP used to instrument NCC was also an eQTL for this gene (pg 24 lines 496-499):

“Third, while we were able to perform sensitivity analyses for ADRB1 inhibition by restricting instruments to SNPs that were also eQTLs for ADRB1, we were unable to find evidence that the SNP used to instrument NCC (rs35797045) was also an eQTL for the gene encoding this target.”

3.      Also, I think the authors need to explain more about the logic of using MR here. The exposures seem to be the drug targets (ACE / ADRB1 / NCC inhibition I think), and the instruments are the selected SNPs. For running MR, we need to have estimated associations between the instruments and exposure, here the ACE / ADRB1 / NCC inhibition.  However, based on my understanding, there seems to be no direct observation of ACE / ADRB1 / NCC inhibition and no GWAS data directly for these drug targets. So instead of using estimated associations between the instruments and exposure, the authors seem to have used associations between the instruments and SBP to run MR. If my understanding is correct, then that is not a standard use of MR as the associations between instruments and the exposure are missing. Thus, more thorough discussions are needed to explain why using associations between the instruments and SBP make sense and what are the underlining assumptions.

Response: We used pQTLs to develop instruments for ACE inhibition (i.e. genetic variants robustly associated with circulating levels of ACE). As mentioned above, we did not have data available on protein expression for ADRB1 and NCC so used a “downstream biomarker” approach to instrument inhibition of these targets which has been used previously across drug-target MR analyses. As mentioned in our response to Q#2 from the reviewer, in sensitivity analyses restricting the ADRB1 instrument to SNPs that are also eQTLs for ADRB1 expression, our findings were similar to those that used our primary ADRB1 instrument. We were unable to find evidence that the SNP used to instrument NCC (rs35797045) was also an eQTL for the gene encoding this target which is mentioned in the Limitations. In the Main text we have emphasised that valid causal inference in MR analyses requires three assumptions having been met and that, though steps were taken to examine potential violations of these assumptions (e.g. “positive control” analyses, restriction of instruments to eQTLs for ADRB1, examination of the association of an instrument with previously reported risk factors for a relevant cancer endpoint for ACE), we cannot exclude potential violations of these assumptions. In the main text we also now emphasise that as pQTL data for ADRB1 and NCC becomes available in GWAS, there is merit in exploring whether the findings presented in this manuscript can be replicated using alternate drug-target MR methodologies (as can be seen on pg 25 lines 534-537):

“As data on circulating levels of ADRB1 and NCC become available in future GWAS, there would be merit in evaluating whether findings presented in this analysis can be replicated when using alternate instruments developed from protein quantitative loci for these targets.”

4.      On page 10, the method "Inverse-variance weighted random -effects models" need a citation. Is this the IVW method proposed in Burgess and Thompson 2013? What does the "random-effects" part refer to?

Response: We thank the reviewer for highlighting this citation omission when mentioning the IVW random-effects model. We have now included a citation when mentioning this model (Bowden et al. A framework for the investigation of pleiotropy in two‐sample summary data Mendelian randomization. Stat Med. 2017 May 20; 36(11): 1783–1802). We now mention that random-effects permit heterogeneity in causal estimates in an IVW model (pg x line Y):

“Inverse-variance weighted random-effects models (permitting heterogeneity in causal estimates) were employed to estimate causal effects of genetically-proxied drug target inhibition on cancer risk”

5.      For the section "Colon transcriptome-wide Mendelian randomization analysis", can the authors explain in what sense are the analyses done in this section Mendelian randomization analysis?

Response: We agree with the reviewer that “Mendelian randomization” is not the correct way to describe the analyses performed in this section because formal instrumental variable analyses were not performed. We now describe these analyses as “Genetic risk score analyses” throughout the main text and supplementary materials.
